# Medicinal plants: bioactive compounds, biological activities, combating multidrug-resistant microorganisms, and human health benefits - a comprehensive review

**DOI:** 10.3389/fimmu.2025.1491777

**Published:** 2025-04-28

**Authors:** Mohamed T. El-Saadony, Ahmed M. Saad, Dina Mostafa Mohammed, Sameh A. Korma, Mohammad Y. Alshahrani, Ahmed Ezzat Ahmed, Essam H. Ibrahim, Heba M. Salem, Samar Sami Alkafaas, Abdullah M. Saif, Sara Samy Elkafas, Mohamed A. Fahmy, Taia A. Abd El-Mageed, Mariam M. Abady, Hanya Y. Assal, Marawan K. El-Tarabily, Betty T. Mathew, Synan F. AbuQamar, Khaled A. El-Tarabily, Salam A. Ibrahim

**Affiliations:** ^1^ Department of Agricultural Microbiology, Faculty of Agriculture, Zagazig University, Zagazig, Egypt; ^2^ Biochemistry Department, Faculty of Agriculture, Zagazig University, Zagazig, Egypt; ^3^ Nutrition and Food Sciences Department, National Research Centre, Giza, Egypt; ^4^ Department of Food Science, Faculty of Agriculture, Zagazig University, Zagazig, Egypt; ^5^ Department of Clinical Laboratory Sciences, College of Applied Medical Sciences, King Khalid University, Abha, Saudi Arabia; ^6^ Biology Department, College of Science, King Khalid University, Abha, Saudi Arabia; ^7^ Blood Products Quality Control and Research Department, National Organization for Research and Control of Biologicals, Cairo, Egypt; ^8^ Department of Poultry Diseases, Faculty of Veterinary Medicine, Cairo University, Giza, Egypt; ^9^ Department of Diseases of Birds, Rabbits, Fish & their Care & Wildlife, School of Veterinary Medicine, Badr University in Cairo (BUC), Cairo, Egypt; ^10^ Molecular Cell Biology Unit, Division of Biochemistry, Department of Chemistry, Faculty of Science, Tanta University, Tanta, Egypt; ^11^ Division of Biochemistry, Department of Chemistry, Tanta University, Faculty of Science, Tanta, Egypt; ^12^ Faculty of Control System and Robotics, Information Technologies, Mechanics and Optics University, Saint-Petersburg, Russia; ^13^ Production Engineering and Mechanical Design Department, Faculty of Engineering, Menofia University, Menofia, Egypt; ^14^ Soils and Water Science Department, Faculty of Agriculture, Fayoum University, Fayoum, Egypt; ^15^ Department of Bio-Analytical Science, University of Science and Technology, Daejeon, Republic of Korea; ^16^ Faculty of Biotechnology, October University for Modern Sciences and Arts, 6^th^ October City, Egypt; ^17^ Faculty of Medicine, University of Debrecen, Debrecen, Hungary; ^18^ Department of Biology, College of Science, United Arab Emirates University, Al Ain, United Arab Emirates; ^19^ Food Microbiology and Biotechnology Laboratory, Food and Nutritional Science Program, North Carolina A&T State University, Greensboro, NC, United States

**Keywords:** antibiotics, antimicrobial agents, herbal medicine, human health, mechanism of action, multidrug resistance, pathogens

## Abstract

In recent years, medicinal plants have gained significant attention in modern medicine due to their accessibility, affordability, widespread acceptance, and safety, making herbal remedies highly valued globally. Consequently, ensuring medicinal plants’ quality, efficacy, and safety has become a critical concern for developed and developing nations. The emergence of multidrug-resistant microorganisms poses a serious global health threat, particularly in low-income regions, despite significant advancements in antimicrobial drugs and medical research over the past century. The rapid spread of these multidrug-resistant infections is primarily attributed to improper prescriptions, overuse, and unregulated access to antibiotics. Addressing these challenges, the standardization of plant-derived pharmaceuticals could pave the way for a transformative era in healthcare. Preserving and leveraging the historical knowledge of medicinal plants is essential before such valuable information is lost. Recently, there has been growing interest among natural and pharmaceutical scientists in exploring medicinal plants as potential sources of antimicrobial agents. This current review aims to identify the most common pathogens threatening human health, analyze the factors contributing to the rise of drug-resistant microorganisms, and evaluate the widespread use of medicinal plants across various countries as alternative antibiotics, highlighting their unique mechanisms of antimicrobial resistance.

## 1 Introduction

In 1928, Alexander Fleming serendipitously discovered penicillin, the first natural antibiotic, marking the onset of antibiotic resistance in the early 20^th^ century (1). This initial discovery catalyzed the advancement of further antibiotics, including streptomycin, chloramphenicol, erythromycin, and chlortetracycline, resulting in a “golden era” of antibiotic development from 1960 to 1980 (2). Nonetheless, the goals of the pharmaceutical sector have changed, resulting in a decrease in the discovery of novel antibiotics and an increase in antimicrobial resistance (AMR) (3). As a result, drug-resistant bacterial infections currently represent a considerable worldwide health risk (3, 4). The comprehensive antibiotic resistance database (CARD) includes more than 5,000 resistance sequences, with a restricted subset linked to notable diseases of concern (4). The problem is exacerbated by the slow pace of new effective antibiotic discovery (5).

AMR is a natural phenomenon in which microorganisms (bacteria, fungi, and protozoa) acquire the capability to endure and proliferate despite the administration of medications such as antibacterial, antifungal, and antiprotozoal antibiotics (6). Antibiotics are essential for addressing bacterial illnesses; however, their extensive application, particularly in resource-limited environments, generates selective pressure that fosters the development of resistance (7, 8). This results in heightened morbidity and death (8). The emergence of “superbugs,” which are resistant to many medications, highlights the gravity of the issue, leading the World Health Organization (WHO) to designate AMR as a significant global health challenge (7). Recent evidence reveals that AMR currently accounts for millions of fatalities annually, with forecasts indicating a substantial rise to 10 million deaths per year by 2050 (9).

The rise of antibiotic resistance poses a significant threat to global health, with methicillin-resistant *Staphylococcus aureus* (MRSA) serving as a prime example of a “superbug” contributing substantially to mortality from drug-resistant infections (10). Bacteria, among the earliest life forms on Earth, are ubiquitous, inhabiting both domestic and professional environments. While most bacterial species are harmless to humans, specific strains, including *S. aureus, Helicobacter pylori, Escherichia coli*, and *Bacillus anthracis*, can overcome host defenses and induce severe illnesses (10, 11). These infections can present as various diseases, including pneumonia, endocarditis, septicemia, and osteomyelitis, underscoring the broad pathogenic capabilities of these organisms (11, 12).

Healthcare-associated infections (HAIs) represent a persistent challenge to patient safety and public health, often leading to serious complications and placing a considerable burden on society (13). Conventional methods for preventing clinical infections have focused on aseptic techniques and systemic antibiotic treatments (13). However, these methods frequently fail to combat established infections effectively (14). A particularly striking example of this challenge is the treatment of infections associated with medical devices. Systemic antibiotic therapy for infections linked to devices such as catheters, artificial prosthetics, subcutaneous sensors, and orthopedic implants demonstrates a disappointingly low success rate, ranging from 22% to 37% (15). The restricted effectiveness highlights the challenge of eliminating established infections when foreign elements are present since they might facilitate bacterial colonization and biofilm development (15).

Furthermore, administering high doses of antibiotics, often necessary to treat localized infections, can harm surrounding tissues. Such high concentrations can lead to cytotoxicity and adverse reactions, further complicating treatment and potentially hindering patient recovery (16). The misuse of antibiotics may be even more alarming due to its contribution to the acceleration of bacterial drug resistance development and dissemination (17). The selective pressure exerted by frequent antibiotic exposure allows resistant strains to thrive, gradually diminishing the effectiveness of these crucial medications (17). This establishes a detrimental loop in which infections become progressively difficult to manage, hence intensifying the demand for elevated antibiotic dosages and aggravating the issue of resistance (17). The convergence of these factors - the rise of superbugs such as MRSA, the difficulties in treating device-related infections, and the adverse effects of high-dose antibiotic therapy - highlights the critical need for novel strategies to prevent and manage bacterial infections in the face of rising AMR (17).

Despite advancements in developing novel antimicrobial agents to address drug-resistant bacteria, such as antibacterial peptides, amphiphiles, and antimicrobial materials, including nanoparticles, hydrogels, engineered surfaces, and coatings, bacterial resistance continues to pose a substantial challenge (18). Current research efforts are, therefore, increasingly focused on developing strategies that can effectively eliminate bacteria without simultaneously driving the evolution and spread of further resistance (18). Consequently, it is imperative to identify novel antimicrobial treatment agents. In recent years, scientific and pharmaceutical communities have exhibited an interest in medicinal plants as potential sources of antimicrobial drugs. Employing selective screening of phytochemicals through medical data offers a dependable approach for identifying innovative medicines (19).

Chinese researchers identified artemisinin from *Artemisia annua* utilizing insights from traditional Chinese medical literature (20). This discovery has already led to the rescue of millions of individuals suffering from malaria (21). The WHO currently recommends a combination medication based on artemisinin as the treatment for this highly fatal disease. Currently, this medication is extensively utilized worldwide (22). Due to their historical effectiveness as anti-infective agents, plants historically employed for medicinal purposes may be crucial in identifying innovative therapeutics for diverse microbial illnesses (23–25). Ancient herbal treatments have been used for centuries to ease disorders and improve general well-being (26, 27). Typically, medicinal plants’ leaves, bark, roots, and flowers are amalgamated to produce an infusion (28, 29).

For example, compounds from the family Zingiberaceae, such as turmeric (*Curcuma longa*), and tamarind (*Tamarindus indica*), have been used to cure several diseases caused by pathogenic microorganisms, including diarrhea and dysentery (30, 31). Few studies have investigated the anti-infective properties of medicinal plants, despite numerous recent ethnobotanical surveys indicating their use by individuals to mitigate infectious ailments (32, 33). Many other studies have conducted targeted studies on the beneficial effects of compounds derived from medicinal plants, such as anti-*Candida* agents (34), anti-biofilm agents (35), and inhibitors of resistant microbial isolates (36).

The current review comprehensively examines the multidrug-resistant (MDR) microorganisms that present the greatest threat to human health. It analyzes the various causes contributing to antibiotic resistance and emphasizes the potential of medicinal herbs as a safe and efficient treatment.

## 2 Antimicrobial resistance

In recent decades, microorganisms have increasingly resisted commonly used antibiotics (37, 38). Current medical concerns include not just infectious diseases such as avian influenza, human immunodeficiency virus (HIV), and severe acute respiratory syndrome (SARS) but also the emergence of resistant microorganisms that cause diseases that were previously eradicated, such as tuberculosis and malaria (39). In the past thirty years, the ineffectiveness of antibiotics and the absence of effective vaccinations have led to the demise of more than 25 million individuals, including over 5 million children (7).

**Figure 1:**
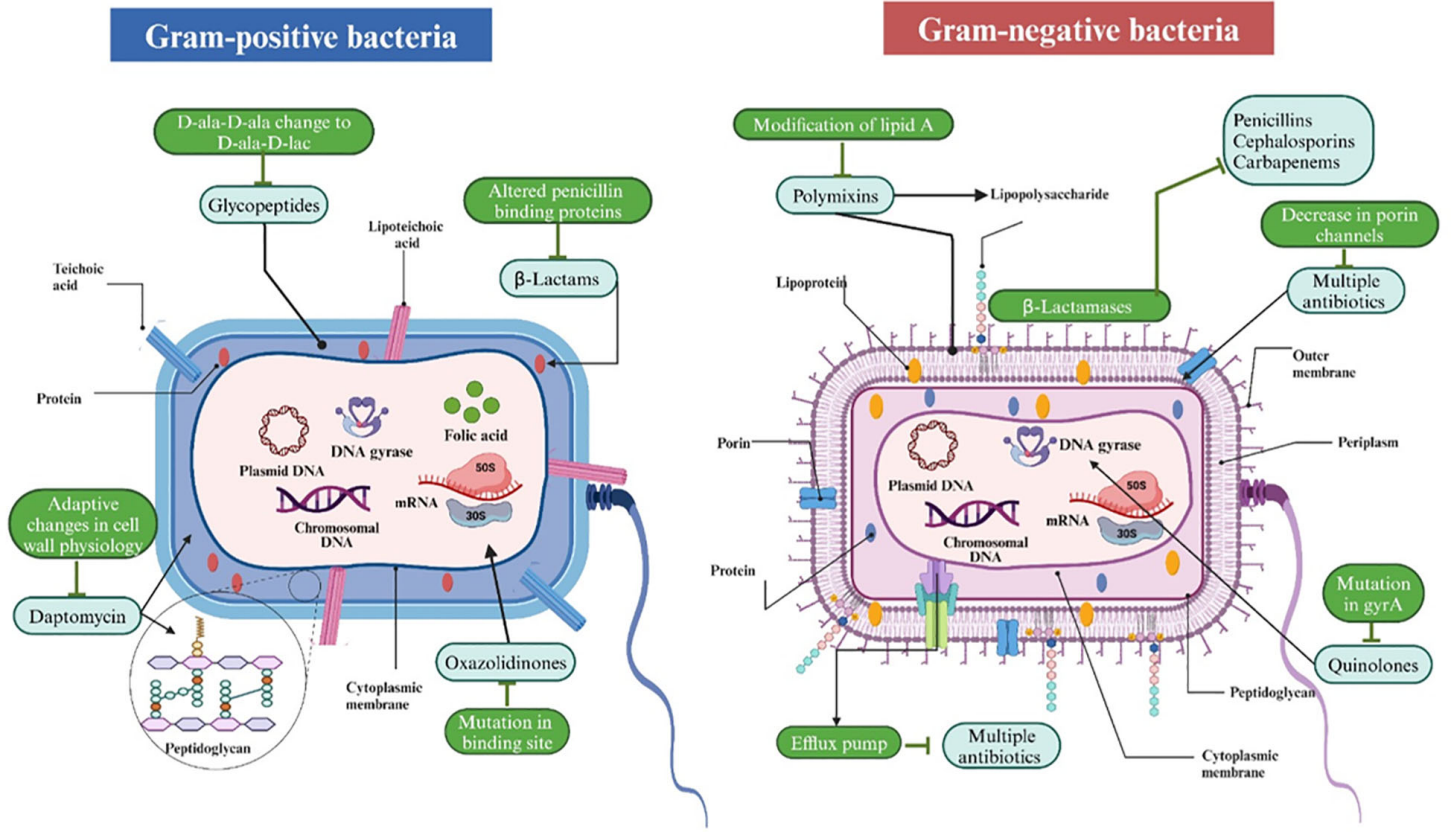
The mechanisms of action of several antibiotics on Gram-positive and Gram-negative bacteria. Gram-positive bacteria, characterized by a thick peptidoglycan layer and teichoic acids, are vulnerable to antibiotics such as glycopeptides (e.g., vancomycin) that impede cell wall formation and daptomycin, which affects membrane potential. Gram-negative bacteria, characterized by an outer membrane and a thinner peptidoglycan layer, exhibit lower permeability yet remain susceptible to polymyxins that compromise the outer membrane and other agents that affect ribosomes (protein synthesis) or DNA gyrase (DNA replication). Both types exhibit analogous vulnerabilities in protein synthesis, folic acid metabolism, and DNA replication, which are targeted by antibiotics such as tetracyclines, sulfonamides, and quinolones, respectively. Resistance mechanisms are also demonstrated, including modified penicillin-binding proteins, beta-lactamase synthesis, efflux pumps, and porin alterations.

Antibiotic resistance occurs when a bacterium becomes unresponsive to previously effective medications. Several frequently utilized antibiotics are now ineffective against 70% of the bacteria that cause nosocomial infections (40). Numerous mechanisms of medication resistance to various illnesses have been proposed, especially for the most commonly utilized pharmaceuticals (40). The modes of action of several antibiotics on Gram-positive and Gram-negative bacteria are illustrated in **Figure 1**.

**Figure 2:**
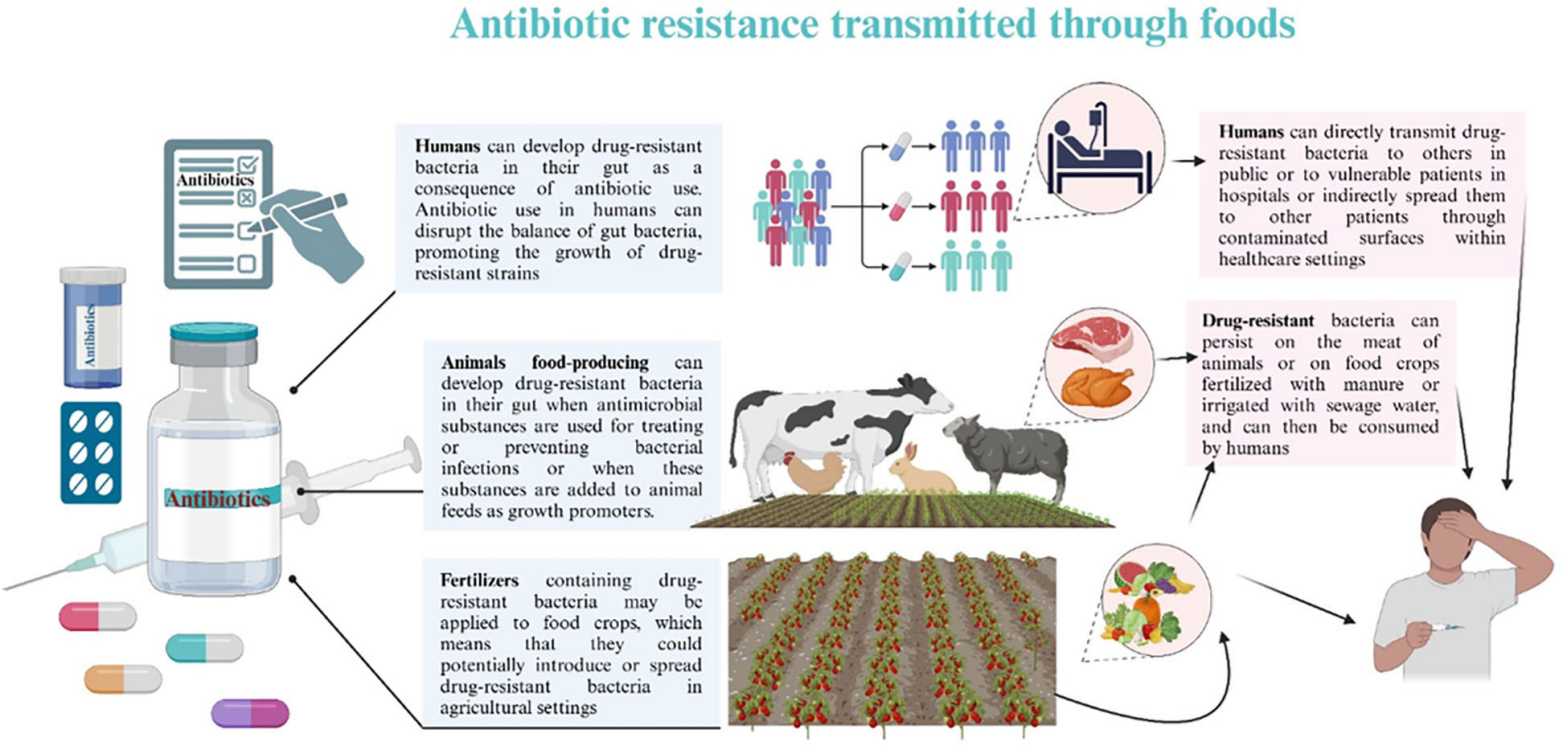
Antibiotic resistance spread through foods. Animal-derived foods, such as dairy, meat, and poultry, provide the principal source of most foodborne microbial diseases. The improper use of antimicrobial agents in animal feed is the principal factor contributing to the increasing public health issue about multi-drug resistant (MDR) microorganisms in these foods. The application of antibiotics in agriculture, livestock production, and human healthcare exerts selection pressure on genes that impart antibiotic resistance. The over-application of antibiotics in animal farming as growth promoters significantly contributes to the development and proliferation of antibiotic resistance. MDR microorganisms, antibiotic residues, and resistance genes can enter agricultural soils via sewage sludge and animal manure, ultimately infiltrating the crop microbiome and the human food chain. Ingesting food can transmit these resistance genes into the human microbiome, posing a significant public health threat.

The improper use of antibiotics has been demonstrated to increase the development and spread of drug-resistant microorganisms (41). Inadequate systems for ensuring the quality and continuous supply of medications, coupled with ineffective surveillance and monitoring mechanisms, along with insufficient control and preventive measures, have substantially facilitated the rise of antibiotic resistance, especially in developing nations where policies are deficient (41). Comprehending how these detrimental organisms evade treatment is essential for formulating alternatives or preserving the effectiveness of current antimicrobial therapies. The spread of antibiotic resistance through foods is depicted in **Figure 2**.

## 3 Factors influencing the development of microbial resistance against antibiotics

Genetic alterations can result in the development of antibiotic resistance by mechanisms such as point mutations, deletions, insertions, or the horizontal transfer of resistant genes (42). Moreover, it has been hypothesized that phenotypic drug resistance may arise concurrently with genetic alterations (42). For an antibiotic to be effective, it must possess the capability to penetrate the cell membrane. Consequently, whatever alterations the bacteria induce in these areas will enhance their resistance to treatments (42).

**Figure 3:**
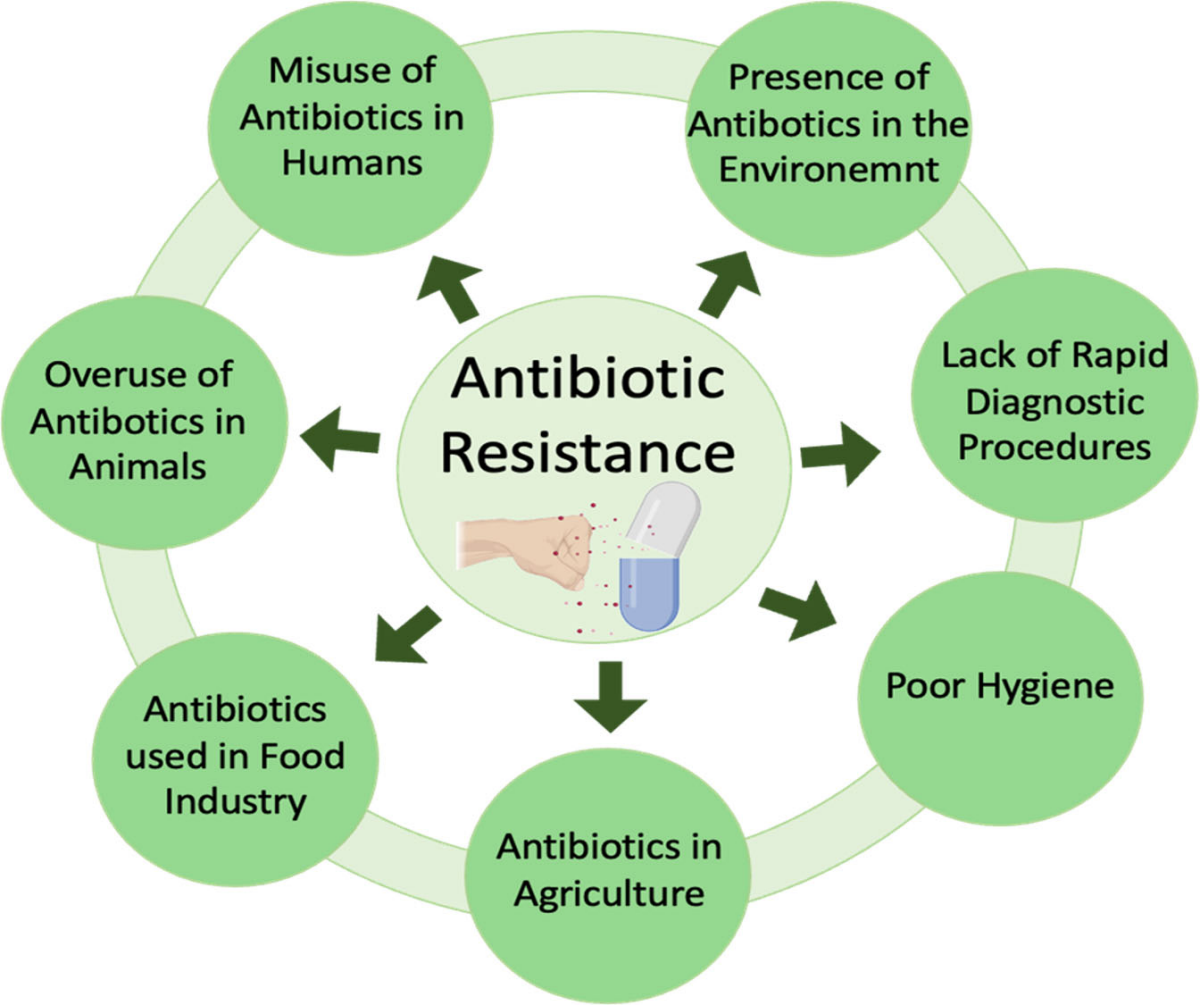
Main factors leading to antibiotic resistance.

Bacteria can affect antibiotic permeability in many ways, including modifying lipopolysaccharides and obstructing molecular interactions with the drug. Alternative mechanisms encompass the development of membrane vesicles, which increases membrane surface area, hence diminishing the amount of medication entering the cell; alterations in antibiotic transporters (porins); and the utilization of efflux pumps to expel the antibiotic from the cytosol (43). **Figure 3** depicts a detailed review of the factors contributing to microorganism antibiotic resistance.

### 3.1 Genetically based drug resistance

Drug resistance in pathogenic microorganisms may be intrinsic or acquired. Foreign-resistant genes can be introduced into the recipient organism by transformation, transduction, or conjugation to confer resistance (43).

### 3.2 Mutations

Mutations at many chromosomal locations cause this AMR and can take the following forms: i) spontaneous mutations: this type of mutation often occurs in actively dividing cells, thus the name growth-dependent mutations (43). They arise as random replication mistakes or improper DNA damage repair (43); (ii) hypermutators: these are highly mutable bacteria, and their hypermutable condition results from prolonged exposure to sublethal antibiotic dosages. These cells can escape the hypermutable stage if they acquire a beneficial mutation and begin to divide and multiply (44); and (iii) adaptive mutagenesis: this mutation happens in slow-growing cells exposed to sublethal antibiotic dosages (43).

### 3.3 Horizontal gene transfer

Horizontal gene transfer allows microorganisms to acquire antibiotic resistance genes from unrelated organisms. This transfer occurs primarily through transformation, transduction, and conjugation. Recombination, a separate process, can also introduce or modify resistance genes within a recipient’s genome (44).

### 3.4 Phenotypic antibiotic resistance

Phenotypic drug resistance, occurring without genetic changes, arises from a microorganism’s physiological state and its inherent survival strategies (45). This resistance can manifest through various mechanisms, including drug indifference, biofilm formation, persistence, inoculum size effects, and metabolic-state-dependent alterations in antibiotic susceptibility (45).

### 3.5 Drug indifference

Susceptibility to antimicrobial agents can vary depending on an organism’s physiological state (46). For instance, dormant cells have demonstrated resistance to ampicillin and tetracycline, whereas rapidly dividing cells are more vulnerable. Conversely, while not actively growing, stationary-phase cells are susceptible to ciprofloxacin and streptomycin (46).

### 3.6 Persistence

The punctate age of dormant cells reveals the occurrence in an antibiotic-resistant microbial culture (47). Persistence comes in two different varieties: Type I and Type II. The first kind is induced by cellular deprivation, whereas the second arises due to the developing cells’ innate biostability (48).

### 3.7 Biofilms

Biofilm development results from attaching free-living (planktonic) cells to surfaces, as detailed below (49): (i) it involves attaching free-living cells to a pre-conditioned surface by random propulsion, chemotaxis, or motion (49). The initial cell attachment is often reversible and is influenced by various physical and chemical variables (50); (ii) The production of adhesion proteins, such as fimbriae, helps in the permanent attachment of the cells. In addition, internal linkages and extracellular polymeric substances (EPS) emerge after cell adhesion (51). The EPS is formed of polysaccharides, such as colonic acid, chitosan, and alginate, typically generated by the microbial cells and serves as a covering for the sessile cells. Some enzymes, RNA, DNA, nutrients, proteins, and surfactants are additional EPS components (51).

During this stage, the biofilm community experiences microbial proliferation and communication using chemical signaling, leading to micro-colonies growth (52). The biofilm’s resistance to some antimicrobials results from its capacity to impede the antibiotics’ penetration via the EPS layer. For example, *Pseudomonas aeruginosa* biofilms are resistant to aminoglycosides (often positively charged) due to the organisms’ alginate secretion, which binds to the positively charged antibiotic (53).

### 3.8 Drug resistance and inoculum size

The efficacy of antibiotic treatment is supposedly affected by inoculum size (54). The elevated importance of the infectious dose of microorganisms *in vivo* may elucidate the disparity between an antibiotic agent’s minimum inhibitory concentration (MIC) *in vitro* and *in vivo* (54). An increased quantity of bacteria that generate enzymes capable of degrading drugs correlates with an escalation in the degradation of medications. The consequence for species devoid of these degrading enzymes remains uncertain (54).

Two factors cause the AMR in these bacteria: (i) a drop in drug concentration resulting from the existence of supplementary drug target sites in both viable and non-viable cells, and (ii) an inadequate ratio of medicinal compounds to a high concentration of microorganisms (55). Based on experimental and mathematical models, both outcomes could apply to drug resistance depending on the size of the inoculums (56).

## 4 Examples of some common MDR microorganisms and their effects on human health

Infectious diseases contribute to around 15% of the 57 million fatalities globally each year, considered to be one of the 21^st^ century’s critical worldwide human health challenges (57). Infectious diseases continue to be the top causes of death and morbidity, accounting for almost 2.2 million deaths among school-aged children and adolescents in 2019. This is particularly true in countries with low and medium incomes (58). Bacteria, fungi, parasites, and viruses cause human infectious illnesses. In underdeveloped nations, the frequency of microbial infections that cause diarrhea, vomiting, respiratory disorders, urinary tract infections, and hospital-acquired diseases is still high (57).

Individuals with immunodeficiency disorders are susceptible to transforming commensal bacteria into pathogens. *Candida albicans, Enterococcus faecalis, E. coli, P. aeruginosa, S. aureus*, and others have been identified as the most common bacteria and yeast responsible for this infection (57). In addition to causing illnesses, *E. coli* and *S. aureus* pose a significant challenge in underdeveloped countries due to their ability to acquire drug resistance (57, 58).

The revival and spread of microbiological infections may be attributed to impoverishment, illiteracy, insufficient sanitation, limited supply of treatments, and deficient health care systems (59). Emerging illnesses and MDR microorganisms have exacerbated the prevalence and severity of infectious diseases worldwide (60). MDR tuberculosis, vancomycin-resistant *S. aureus*, and enterococci are drug-resistant human infections threatening worldwide public health (61). Given these findings, adequate mitigation strategies must be implemented in the healthcare and public health sectors (61). The Centers for Disease Control and Prevention has proposed numerous solutions, including prevention, increased surveillance, and the development of novel treatments such as antimicrobials derived from natural sources (61).

A microbial infection occurs when a microorganism can enter a host’s tissue, damage cells, and cause illness or death by avoiding detection by the host’s defenses, releasing substances into the bloodstream that facilitate further invasions (such as toxins), and exhibiting other clinical symptoms (62). The skin and mucosal surfaces contain many microorganisms contributing to the human body’s natural flora. They often aid the host by countering possible infections at attachment sites and nutrition and producing harmful antimicrobial chemicals against bacteria (62).

Infectious diseases may be transmitted via several means, such as direct contact between individuals, transfer through the respiratory system, contact with mucous membranes or through sexual activity, injection into the bloodstream, transmission by insects, and contact with contaminated objects (62).

Despite substantial efforts in control and prevention, infectious illnesses persist as the primary cause of disease and mortality worldwide (63). About 9.5% of adult deaths per year are attributed to tuberculosis and lower respiratory diseases (63). Furthermore, children under the age of five are disproportionately affected by contagious diseases, including acute respiratory infections, diarrhea, measles, malaria, and HIV/AIDS, which together contribute to 30% of the total yearly death rate (63). The fast growth of MDR microorganisms has been related to the abuse and overuse of antimicrobial agents with extended-release formulations used to treat illnesses (64). Failure to adhere to prescribed antibiotics elevates the risk of developing antibiotic-resistant pathogens, chronic illnesses, and mortality (65). Second- or third-line drugs, typically more expensive and potentially hazardous, are utilized when resistance to first-line antibiotics develops prevalent (65, 66).

The misuse of antibiotics, along with the swift emergence of microbiological illnesses, is further affected by the following aspects (64): (i) Demographic transitions have led to a growing number of vulnerable populations, particularly the elderly, necessitating hospital-based interventions that expose patients to prevalent drug-resistant pathogens, thereby jeopardizing their health; (ii) Urbanization contributes significantly to the proliferation of diseases such as typhoid, tuberculosis, respiratory infections, and pneumonia, exacerbated by overpopulation and unsanitary conditions; (iii) Environmental degradation, pollution, and variable climatic conditions influence the incidence and transmission of diseases, particularly those vectored by insects, such as malaria; (iv) The AIDS epidemic has markedly increased the population of immunocompromised individuals susceptible to various illnesses; and (v) Annually, millions of cases arise from drug-resistant infectious diseases, including tuberculosis and malaria, due to their recurrent nature (64). Most of this cost is shouldered by low-income countries, mostly due to their insufficient social investments in infrastructure, educational access, training, and other resources necessary for managing and mitigating the development of drug resistance (63, 65, 66).

### 4.1 E. faecalis

Enterococci are facultative anaerobes, ovoid (0.5–1 µm in diameter), stationary, and non-sporulating, Gram-positive cocci generally regarded as common human commensals (67). They are exceptionally adapted to prosper in nutrient-rich environments that are deficient in oxygen, such as the diverse ecosystems present in the oral cavity, gastrointestinal tract, and vaginal canal (68). The typical quantity of bacterial cells excreted is roughly 10^11^ per gram of feces. They predominantly constitute the commensal gut microflora (69).

Although there are over 40 physiologically unique species of *Enterococcus*, only two species account for over 90% of enterococci infections: *E. faecalis*, and *E. faecium* (67). Infrequently, enterococci induce infections in healthy and consistent hosts. Endocarditis, sepsis, surgical wound illnesses, urinary system diseases, secondary bacteremia, and inflammatory bowel diseases are among the nosocomial ailments to which *E. faecalis* is reportedly becoming a significant contributor, according to surveillance data (70).

Enterococci species’ pathogenicity is determined by several characteristics, such as their typical habitat of the gut, how easily they increase there, the ability to detect a range of extracellular matrix proteins, and the availability of collecting material (71). Enterococci rely on biofilm formation to facilitate the development of endodontic and urinary system infections and endocarditis. Sortase C gene, EBPa, EBPb, and EBPc are the components of endocarditis and biofilm-associated pili (EBP) (72). *E. faecalis* secretes virulence factors that actively suppress host immune responses, including the production of toxins such as cytolysin and extracellular enzymes (73). Antibiotic resistance in enterococci increases the likelihood of colonization and infection and is directly associated with the therapeutic importance of the bacteria. All enterococci have reduced sensitivity to ampicillin, cephalosporins, penicillin, and all semisynthetic penicillins due to the production of penicillin-binding proteins with poor affinity (73).

One of this adaptation’s most obvious effects is the emergence of the genes that cause glycopeptide-vancomycin resistance (74). During the 1960s, MRSA emerged and spread in correlation with the use of vancomycin (74). Conversely, unlike MRSA, enterococci can assemble and sustain a range of gene clusters encoding the metabolic apparatus required for vancomycin resistance (74). Antibiotic-resistant enterococci can transfer their resistance to other pathogens, such as *S. aureus* and antibiotic-susceptible enterococci, by pheromone-mediated conjugative plasmids or transposons. This significantly threatens public health (74).

Recombinant transmission of dynamic genetic components requires interaction between the donor and recipient cells (74). The interaction occurs inside the microbial biofilm and is encouraged by the release of bacterial sex pheromones, which are small peptides that induce a sexual response and lead to the aggregation or clustering of cells (74). Up to 90% of human enterococcal infections are caused by *E. faecalis*, which also ranks third among hospital-acquired diseases brought on by resistant bacteria. For these reasons, *E. faecalis* is a serious hazard to human health (74).

### 4.2 S. aureus

*S. aureus* is a Gram-positive nonmotile bacterium which does not produce spores. The diameter of *S. aureus* ranges from 0.5 to 1.5 µm (75). It constitutes a component of the standard microbial population within the human body, namely in the anterior region of the nostrils, and has the potential to induce infections in individuals with compromised immune systems (75). Approximately 30% of individuals are permanent carriers of this bacterium. *S. aureus* has a resilient, somewhat amorphous protective coating measuring 20–40 nm in thickness on its cell wall (76). Peptidoglycan, constituting 50% of the staphylococci’s mass, is crucial for forming the thick, multilayered structure of the cell wall and sustaining the elevated intracellular osmotic pressure of the bacterium (77, 78).

When the host’s immune systems are compromised, colonization increases the probability of infection as the pathogen becomes virulent (79, 80). *S. aureus* is a significant contributor to both hospital-acquired and community-acquired infections, which can result in serious consequences if not addressed. The excessive use of antibiotics intensifies this organism’s resistance to drugs (81, 82).

*S. aureus* infects by breaching the integrity of the skin or mucous membranes, disseminating locally or systemically to distant organs, leading to severe and life-threatening invasive infections, including bloodstream infections, pneumonia, systemic infections, joint infections, and osteomyelitis (83). It also induces toxin-mediated illnesses when ingested through contaminated food. Food poisoning, and toxic shock are toxin-mediated illnesses caused by *S. aureus* (84, 85). In certain circumstances, exposure to secretions such as saliva or aerosols emitted during sneezing or coughing may facilitate disease transmission. This bacterium may also be present in unpasteurized milk and other animal products (86).

Pigs, cattle, and poultry may contract *S. aureus*, resulting in mastitis, arthritis, septicemia, and other conditions (87). The virulence factors of *S. aureus* are defined by their mechanisms of invasion and proliferation, production of extracellular substances, toxins, and capacity to form biofilms (88, 89). Infection pathogenesis often commences with cellular attachment, succeeded by bacterial invasion and colonization of host tissue. This process releases many adhesion proteins, including elements of the microbial surface that recognize sticky matrix molecules (90, 91).

In contrast to virulence proteins associated with the cell wall, the secreted virulence factors of *S. aureus* aggressively undermine human defenses by causing harm to host cells and tissues (91). They also impair the host’s immune response by releasing nutrients and promoting the dissemination of pathogens (92). Released virulence factors are classified into four primary categories: superantigens, diverse exoenzymes, random proteins, and pore-forming toxins (88, 93).

Research has shown that staphylococci can acquire resistance to antibiotics such as erythromycin, ampicillin, tetracycline, methicillin, and vancomycin (94). The van gene confers resistance to vancomycin, likely resulting from gene transfer between *S. aureus* and vancomycin-resistant enterococci (94). The swift global proliferation of methicillin- and vancomycin-resistant *S. aureus* strains is rendering containment increasingly unfeasible, potentially reverting us to the pre-antibiotic era (94). In regions with minimal methicillin resistance, penicillin, β-lactam antibiotics, cephalosporins, and clindamycin continue to be effective in treating *S. aureus* infections (95). The utilization of non-lactam antibiotics for the treatment of *S. aureus* infections has markedly increased owing to the emergence of antibiotic-resistant bacteria, especially MRSA (96).

Methicillin resistance in *S. aureus* developed swiftly in 1961, within two years after the drug’s clinical launch (97). This bacterium is intrinsically vulnerable to β-lactam agents that impede cell wall synthesis by binding to proteins implicated in peptidoglycan assembly (98). MRSA exhibits resistance to β-lactam antibiotics, including methicillin, isoxazolyl penicillin, and cephalosporins, by modifying its target site. The modification is achieved through the acquisition of penicillin-binding protein 2a, produced by the mecA gene (99). Nosocomial infections, including MRSA, have been acknowledged for numerous years (100). This bacterium can colonize and establish biofilms on biomaterials, leading to a significant prevalence of hospital-acquired infections referred to as HA-MRSA. HA-MRSA is a kind of MRSA infection identified when a positive culture is obtained more than 48 h after a patient’s hospital admission. This infection is managed in a hospital environment (21).

Community-acquired MRSA refers to the transmission of MRSA to the broader community. Various populations, including athletes, inmates, prospective military personnel, nursery attendees, injectable drug users, those often exchanging contaminated commodities, and residents of densely populated areas, have been associated with community-acquired MRSA epidemics (101, 102). Individuals with MRSA who come into contact with animals may transmit the bacterium, leading to livestock-associated infections (LA-MRSA). LA-MRSA has been identified in many animal species (103).

### 4.3 S. epidermidis

*S. epidermidis*, a member of the human body’s normal flora, is frequently isolated from various epithelia, including the nose, axillae, and head (104). This organism normally has a symbiotic relationship with its host and prevents pathogenic microorganisms from invading the host (105). Because of its exploitative qualities, particularly in nosocomial infections in immunocompromised patients, the medical community is very interested in controlling this disease (106). Infections produced by coagulase-negative *S. epidermidis*, possibly related to its proclivity to build biofilms, are prevalent in patients with implanted medical devices (107).

*S. epidermidis* infections can be caused by normal skin flora at the insertion site, contaminated medical equipment prior to implantation, or healthcare professionals (108). It has also been proposed that these infections were caused by long-term hospitalizations, surgical procedures after implantation, infections in neighboring tissues, and tissue damage following implantation operations (108, 109). Methicillin, one of the principal treatments for *Staphylococcus* species infections, is resistant to 75-90% of *S. epidermidis* strains obtained from hospital specimens (110). This bacterium has several antibiotic-resistant forms, including tetracycline, chloramphenicol, erythromycin, sulfonamides, fluoroquinolones, clindamycin, and rifamycin (111).

### 4.4 L. monocytogenes

*L. monocytogenes* is a bacterium that causes illness and belongs to the Gram-positive bacteria found in wet environments such as soil, water, and rotting plants and animals (112). It may thrive with refrigeration and other food preservation techniques. People who consume *L. monocytogenes*-contaminated food may get listeriosis. *L. monocytogenes* is commonly transmitted through contaminated food processing (112). The symptoms of the condition might persist anywhere from a few days to many weeks, depending on its severity. Fever, muscle cramps, vomiting, diarrhea, and nausea are examples of minor symptoms (112). The most severe form of listeriosis is characterized by headaches, stiff neck, disorientation, loss of balance, and convulsions. Listeriosis can kill infants, the elderly, and immunocompromised people (112, 113).

### 4.5 E. coli

*E. coli*, a Gram-negative, rod-shaped, facultatively anaerobic coliform bacterium, is frequently found in the lower intestines of warm-blooded mammals (114). The majority of *E. coli* strains are typical residents of the gastrointestinal tracts of humans and animals. Furthermore, it was demonstrated that *E. coli* cells penetrate the neonatal digestive tract immediately after birth (115). The disease-causing groups are classified into two categories: one responsible for intestinal problems and another for infections beyond the intestines, such as sepsis and bladder infections (116).

*E. coli* is characterized by a diverse array of harmful genotypes, categorized as pathotypes based on their ability to cause disease. Entero-toxigenic *E. coli* (ETEC) strains have been identified in neonates experiencing diarrhea in developing countries, and they are responsible for secretory diarrheal infections in animals (117). Two serotypes of enteropathogenic *E. coli* (EPEC) are present: O55:H6 and O127:H6, with atypical strains inducing diarrhea in developed nations. Conversely, the majority of prevalent strains are obtained by fecal contamination (118). Enterohemorrhagic *E. coli* (EHEC) strains are associated with foodborne infections commonly acquired by the consumption of milk and inadequately cooked animal products, particularly meat (118). Two distinct kinds of Shiga-like toxin producers are found in this group: stx1 and stx2 (119). Entero-invasive *E. coli* (EIEC) have comparable biochemical, genetic, and pathogenic properties with *Shigella* species and are known to cause various human diseases (119). Colitis and watery diarrhea are the most common diseases EIEC causes; these infections differ from those brought on by other strains of the same bacteria (119).

Another variant of *E. coli* is referred to as diffusely adherent *E. coli* (DAEC) strains. These strains are characterized by the diffusely adherent pattern noted in the HEp-2 adherence assay and are implicated in the etiology of watery diarrhea syndrome in adults (120). Enteroaggregative *E. coli* (EAEC) is a strain characterized by its remarkable adhesion to HEp-2 cells in culture. They are associated with chronic diarrhea, notably in underprivileged nations and the developed world, particularly among those with HIV/AIDS (120). Human extra-intestinal infections may also be induced by *E. coli* pathotypes linked to sepsis, meningitis, urinary tract infections, and bloodstream infections. *E. coli* O18:K1:H7 can cause invasive infections in newborns and urinary tract problems. Certain *E. coli* strains exhibit resistance to carbapenems, β-lactams, and various other pharmaceuticals (120).

### 4.6 K. pneumoniae

It is a Gram-negative bacterium that commonly inhabits human lips, skin, and intestines, and has been associated with nosocomial infections. Certain illnesses, such as cancer, diabetes mellitus, liver and biliary tract conditions, and alcoholism, impair the immune system, heightening vulnerability to *K. pneumoniae* infections (121). This bacterium is also known to cause pneumonia, urinary tract infections, skin infections, and open wounds (121).

*Klebsiella* infections are frequently managed with cephalosporins, either alone or in conjunction with aminoglycosides (122). Nonetheless, the extensive administration of powerful antibiotics in individuals with *Klebsiella* infections has resulted in the emergence of drug-resistant bacteria, especially those that produce extended-spectrum beta-lactamase (ESBL) (123). Carbapenems can adversely affect ESBL producers; however, these organisms have evolved novel enzymes known as *K. pneumoniae* carbapenemases (KPCs) that can degrade carbapenems and have disseminated globally (124, 125). No advancements have been made in synthetic or natural antimicrobials, hence ESBL-producing bacteria remain a significant danger. Bacteria that produce ESBL exhibit increasing resistance to antibiotics, including fluoroquinolones, aminoglycosides, tetracyclines, trimethoprim/sulfamethoxazole, and chloramphenicol (126).

### 4.7 P. aeruginosa

It is a potentially pathogenic Gram-negative, rod-shaped, spore-forming, and monotrichous bacterium found in aquatic environments and soil surfaces. It possesses an iridescent appearance and emits an aroma reminiscent of grapes or tortillas (127). It exhibits optimal growth within the temperature range of 25°C to 37°C, and its ability to flourish at 42°C sets it apart from numerous other *Pseudomonas* species. *P. aeruginosa* is a ubiquitous bacterium capable of persisting in various ecological environments (127).

The bacterium’s intrinsic antibiotic resistance is a significant characteristic that enables its survival under many extreme environmental conditions, both natural and artificial, including biofilms in healthcare settings. This bacterium induces nosocomial infections, especially in immunocompromised persons with neutropenia, severe burns, and cystic fibrosis (128).

Pathogenic *P. aeruginosa* can cause external and internal infections, including wounds, urinary tract infections, pneumonia, eye infections, sepsis, and endocarditis (129). A surge in MDR *Pseudomonas* infections has been seen, irrespective of the combination therapy employed (130). The pathogens inflict significant damage to host tissue by cleaving immunoglobulin G and A, modifying the cytoskeletal structure, and disintegrating actin filaments. Specifically, exotoxins, proteases, and exoenzymes, this mechanism facilitates the invasion, spread, and advancement of chronic infections (131).

*P. aeruginosa* poses a significant challenge in the medical domain for the treatment of infections acquired in hospital environments and the population (132). This bacterium can acquire resistance genes by horizontal gene transfer to aminoglycoside-modifying enzymes and extended-spectrum β-lactamases (132). Resistance may also emerge from modifications in chromosomal genes located at the efflux and target sites, and pathogenic isolates of this or related species have been documented to exhibit antibiotic resistance by innate, acquired, and adaptive mechanisms (132). *P. aeruginosa* exhibits resistance to several antibiotics, including cephalosporins, carbapenems, aminoglycosides, quinines, ureidopenicillins, quinolones, cefepimes, penicillin, and polymyxins (133, 134).

### 4.8 *Salmonella enterica* serovar Typhimurium

*Salmonella* species, such as *Salmonella enterica* serovar Typhimurium, Typhi, and Enteritidis, are worldwide dispersed Gram-negative bacteria recognized as significant contributors to several human diseases (135). *Salmonella* can be disseminated through multiple vectors, including contaminated food and water, human and animal excrement, and deceased animals. Also, *S. enterica* serovar Typhimurium can be obtained from numerous infected foods, including beef and pig products (136).

Salmonellosis is also linked to contaminated fresh fruits and vegetables, including apples, cantaloupe, alfalfa sprouts, mangoes, lettuce, cilantro, unpasteurized orange juice, tomatoes, melons, celery, and parsley (137). Poultry products, such as chickens, turkeys, geese, and ducks, have been associated with the transmission of *Salmonella* infections. *Salmonella* exhibiting resistance to ampicillin, chloramphenicol, and trimethoprim-sulfamethoxazole have been detected in multiple regions, particularly *S. enterica* serovar Typhimurium (138).

### 4.9 Shigella flexneri

*Shigella* is a bacterium classified under the Enterobacteriaceae family. It is distinguished by its Gram-negative characteristics, absence of capsules, and lack of motility (139). *S. flexneri* is one of the four species of this genus commonly associated with bacillary diarrhea, the principal clinical manifestation of shigellosis, characterized by inflammation of the colon. The symptoms may range from asymptomatic carriage to diarrhea and dysentery (139).

*Shigella* can reside in humans and captive monkeys, and it is estimated that fewer than 200 bacterial cells are sufficient to induce an infection. Consequently, the proliferation of the bacteria is more probable in highly populated regions with inadequate sanitation, and annual *Shigella* outbreaks are anticipated to surpass 150 million cases (140). Shigellosis predominantly affects children in developing nations, who are the most severely harmed. Conversely, the epidemic is widespread in congested establishments inside affluent countries, including daycare centers, correctional facilities, and military recruitment camps (141).

Despite the manageable prevalence of shigellosis infections due to the availability of several antibiotic regimens, the emergence of drug-resistant strains is alarming. Antibiotics such sulfonamides, tetracycline, ampicillin, and chloramphenicol are ineffective against these pathogens (141).

### 4.10 C. albicans

Identifying appropriate treatment for fungal infections without harming the host is the most difficult aspect. This is attributable to the physiological cellular similarities between fungi and humans. Consequently, effective therapies for fungal infections are limited in comparison to those for bacterial disorders (111). Mycoses have become a notable concern in modern medicine owing to the rising population of patients receiving immunosuppressive treatment or experiencing immunodeficiency (142).

*Candida* species generally exist as unicellular yeasts in a symbiotic connection with their human hosts (143). These yeasts are often confined to the skin, gastrointestinal tract, reproductive organs, and mucosal surfaces of the oral cavity. *C. albicans* can alternate between two unique forms: a unicellular yeast that reproduces via budding and can also produce elongated filaments, comprising pseudomycelium (143). This capability is essential in infections where the invasive hyphae can disseminate throughout the affected tissue or organ (143).

In contrast, yeast cells disseminate to many regions. This commensal yeast is classified within the genus *Candida* and is typically located in the oral cavity, dermis, gastrointestinal tract, and vaginal canal of females (144). *C. albicans* demonstrates a growth pattern marked by yeast cells that are spherical to oval, measuring 3 to 5 µm in width and 5 to 10 µm in length (145).

*C. albicans* often exists as a nonpathogenic commensal organism. Nonetheless, an excessive presence of this yeast may create a perilous scenario as it can inhabit almost all human tissues and provoke severe systemic infections in individuals with compromised immune systems (146). *C. albicans* is the sixth most prevalent pathogen in the bloodstream for both acute and chronic yeast infections in humans and is a recognized source of nosocomial infections, particularly in immunocompromised individuals (147).

HIV infection and diabetes are two comorbid conditions frequently linked to this yeast. It is well acknowledged as the etiology of vulvovaginal and oropharyngeal candidiasis. Oral candidiasis, vulvovaginal candidiasis, and invasive candidiasis are illnesses resulting from *Candida* infections. Oral candidiasis is an opportunistic infection of the oral cavity. It is prevalent among elderly individuals, especially those who utilize dentures (148). This disease is typically transferred to immunocompromised individuals and may signify systemic diseases such as diabetes (149).

In women of reproductive age, particularly those who are HIV-positive, *C. albicans* is responsible for the heightened prevalence of vulvovaginal candidiasis (150). Nosocomial infections may result from invasive candidiasis, or candidemia, which is the most prevalent opportunistic mycosis worldwide (151). Invasive nosocomial infections can present as urinary tract infections, surgical site infections, bloodstream infections (fungemia), catheter-associated skin abscesses, and myocarditis (152).

*C. albicans* possesses several acknowledged virulence factors implicated in disease. *C. albicans* virulence factors bind to endothelial cells in blood vessels and epithelial cells in the respiratory tract, thereby greatly influencing pseudo-hyphae development (152). Surface recognition molecules, hyphal switching, and phenotypic switching facilitate this process. Extracellular hydrolytic enzymes are crucial for the dissemination of microorganisms since they facilitate adhesion, tissue infiltration, invasion, and ultimately, the destruction of host tissue (152).

A common virulence factor that facilitates sickness is hemolysin. Hemolysin-induced erythrocyte lysis enhances iron absorption, an essential component for yeast growth and pathogenic functions (153). *Candida* lysin contributes to immunological activation, phagocyte recruitment, tissue invasion, and epithelial injury (154, 155).

The azole class is the most often used antifungal for the treatment of *Candida* infections (156). They are preferred for managing various *Candida* infections due to their cost-effectiveness, less toxicity, and simple oral administration (156). Currently, there are four primary kinds of antifungal agents utilized for the treatment of severe mycoses, classified according to their mode of action (1): Amphotericin B modifies cell membrane functionality (2); Flucytosine obstructs DNA or RNA synthesis (3); Azoles, including fluconazole, itraconazole, voriconazole, posaconazole, and ravuconazole, impede ergosterol production; and (4) Echinocandins, such as caspofungin, micafungin, and anidulafungin, inhibit glucan synthesis (156, 157).

Over the past decade, there has been a notable rise in the incidence of drug-resistant fungal infections among hospitalized patients. The rise is particularly pronounced with frequently utilized antifungal agents, including fluconazole, miconazole, clotrimazole, tioconazole, amphotericin B, and echinocandins (158). Antifungal resistance mechanisms can be classified into three primary categories: (1) modified drug targets, (2) diminished effective drug concentration, and (3) metabolic bypasses (156).

There are fewer pharmacological options for treating *Candida* infections compared to bacterial or viral infections, and incidences of treatment resistance are more prevalent (159). The initial report of *C. albicans* strains exhibiting resistance to azole antifungals occurred in the late 1980s. Since its discovery in the 1990s, fluconazole has been the preferred antifungal agent for unicellular and multicellular fungal infections (159). Nonetheless, despite its initial effectiveness within the medical community, significant instances of medication resistance have arisen, notably among those who are HIV-positive (160).

### 4.11 Candida tropicalis

*C. tropicalis*, a species separate from *C. albicans*. Non-*C. albicans Candida* (NCAC) is recognized for its ability to induce oral infections (161). The incidence of nosocomial infections, specifically NCAC infections, can be ascribed to several medical interventions and situations, including cancer treatments, surgical implantations, HIV, AIDS, and the overuse of antibiotics (161, 162). *C. tropicalis* is the most pathogenic among the NCAC species due to its capacity to bind and release proteinases. It is one of the most significant invasive features of pathogenic organisms towards epithelial cells (163). It inhabits the mouth cavity and skin and has been associated with esophageal and nosocomial infections (163).

*Candida* strains can form biofilms on medical equipment, making them resistant to antifungal agents (164). The incidence of illnesses attributed to *C. tropicalis* is rising globally, designating this organism as a notable emerging pathogenic yeast. The cause of this organism’s dominance over other NCAC species and its fluconazole resistance remains unidentified (165).

## 5 Medicinal plants as alternative antibiotics

A variety of drugs exist to address infections and their complex antibiotic-resistance mechanisms (166). The mechanism of action of these antimicrobial agents relies on selective toxicity. Studies have shown that medicinal plants contain a diverse assortment of bioactive chemicals, such as coumarins, flavonoids, phenolics, alkaloids, terpenoids, tannins, essential oils, lectins, polypeptides, and polyacetylenes (167). These chemicals serve as essential precursors for antibiotic synthesis (168). Although synthetic antimicrobial agents have received extensive endorsement, natural compounds sourced from various origins such as plants (169, 170), fungi, lichens, endophytes, and marine organisms including seaweeds, corals, and other microorganisms remain a prominent area of investigation (171, 172).

These natural products exhibit significant potential for addressing antibiotic resistance in bacterial pathogens (173). Plant-derived chemicals are notable for their potential in combating bacterial infections. These naturally occurring phytochemicals have demonstrated diverse advantageous qualities, including antioxidant, antibacterial, and antifungal activity (173). They may also significantly improve the effectiveness of current antibiotics, therefore averting more resistance development (174).

The bioactive compounds in medicinal plant extracts can operate through many mechanisms, including interaction with specific bacterial membrane components, such as anionic phospholipids and lipopolysaccharides, leading to bacterial lysis by membrane disruption (175). Hydrophobicity may affect the reaction’s outcome by interacting with the hydrophobic groups within the membrane. Moreover, the plant extract is administered into the bilayer surface through ionic/electrostatic interactions, resulting in the destabilization and rupture of the cellular membrane (46). Furthermore, the aforementioned processes may function when active components contain hydrophobic and hydrophilic residues (175).

### 5.1 Extraction of bioactive compounds from medicinal plants

Bioactive components in plant extracts can be isolated, identified, and evaluated by high-performance liquid chromatography (HPLC) (176). HPLC can be classified into two primary categories: analytical HPLC and preparative HPLC (176). Analytical HPLC primarily assesses the qualitative and quantitative characteristics of a given component. Conversely, preparative HPLC is dedicated to the extraction and purification of a valuable chemical (176). The preparative HPLC method is extensively employed to identify physiologically active chemicals (176).

Mass spectrometry (MS) and nuclear magnetic resonance (NMR) are frequently employed to detect pure substances. MS is a scientific method that use ionization and separation to examine ions according to their mass-to-charge ratio. The mass-to-charge ratio serves as the independent variable for plotting the ion signal in a mass spectrum (176). The elemental or isotopic characteristics of a sample, the masses of particles and molecules, and the chemical structures of molecules can all be determined by MS (176). NMR can accurately detect the chemical features of compounds, including the quantities of carbon and proton atoms, bonding patterns, molecular geometries, and relative and absolute stereochemistry’s (176).

Gas chromatography and mass spectrometry (GC–MS) can be employed to analyze essential oils. This device employs an inert gas stream to introduce the sample into the mobile phase (177). The vaporized sample is upheld by inert material while it traverses the stationary phase of the capillary column. The specific analytes/compounds are transmitted through a MS detector, and the resultant data is presented on a computer or recording apparatus (177).

Utilizing an ethnopharmacological strategy for the screening of bioactive components provides multiple advantages (176). It facilitates the pre-screening of gathered species according to their ethnomedicinal applications and preliminary safety standards, so substantially reducing the time and cost of the process (176, 177). Numerous natural compounds derived from medicinal plants have been effectively identified by the ethnopharmacological method, and these compounds have provided a foundation for innovative methods in the pharmaceutical sector. An example of this is the method employed to extract artemisinin, an antimalarial compound, from the plant *A. annua* (177).

### 5.2 Bioactive compounds from medicinal plants

**Figure 4:**
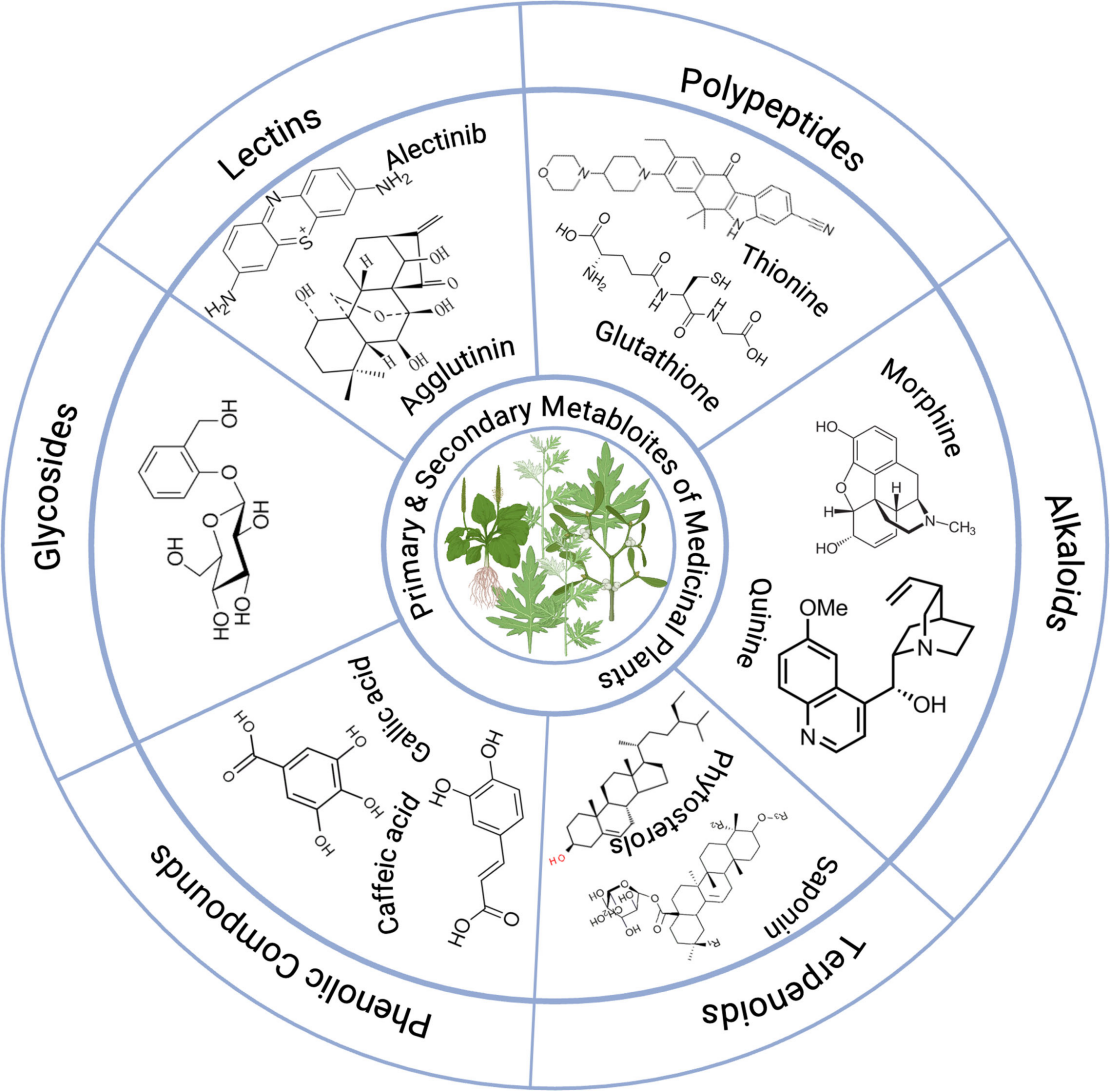
Primary and secondary metabolites of medicinal plants.

The bioactive components of various medicinal plants against certain MDR microorganisms are illustrated in **Table 1**, which demonstrates their effectiveness against specific MDR microorganisms. Furthermore, examples of primary and secondary metabolites found in medicinal plants are presented in **Figure 4**.

**Table 1:** Bioactive compounds of some medicinal plants against certain multi-drug resistant (MDR) microorganisms.

Medicinal plants	Bioactive compounds	MDR microorganisms	References
*Curcuma longa* (turmeric)	Curcumin	*Staphylococcus aureus*	(322)
*Zingiber officinale* (ginger)	Gingerols, and shogaols	*Escherichia coli*	(323)
*Allium sativum* (garlic)	Allicin	*Pseudomonas aeruginosa*	(324)
*Azadirachta indica* (neem)	Azadirachtin	*Klebsiella pneumoniae*	(325)
*Echinacea purpurea* (purple coneflower)	Echinacosides, and flavonoids	*Staphylococcus aureus*	(326)
*Melaleuca alternifolia* (tea tree)	Terpenoids (e.g., Terpinen-4-ol)	*Staphylococcus aureus*, and *Candida*	(327)
*Berberis vulgaris* (barberry)	Berberine	*Escherichia coli*, and *Staphylococcus aureus*	(328)
*Camptotheca acuminata* (happy tree)	Camptothecin	*Escherichia coli*	(329)
*Solanum tuberosum* (potato)	Tomatidine	*Candida albicans*	(330)
*Punica granatum* (pomegranate)	Punicalagins, and ellagic acid	*Aggregatibacter actinomycetemcomitans*	(331)
*Commiphora molmol* (myrrh)	Sesquiterpenes	*Porphyromonas gingivalis*	(332)
*Dicranostigma leptopodum* (eastern horned poppies)	Alkaloids	*Klebsiella pneumoniae*	(333)
*Hypericum perforatum* (St John’s wort)	Hypericin, and flavonoids	*Staphylococcus aureus*	(334)
*Rosmarinus officinalis* (rosemary)	Rosmarinic acid, and carnosic acid	*Escherichia coli*	(335)
*Thymus vulgaris* (thyme)	Thymol, and carvacrol	*Staphylococcus aureus*, and *Escherichia coli*	(336)
*Aloe vera* (cactus)	Aloin, and anthraquinones	*Pseudomonas aeruginosa*, and *Staphylococcus aureus*	(337)
*Ocimum basilicum* (basil)	Essential oils (e.g., linalool)	*Escherichia coli*, and *Staphylococcus aureus*	(338)
*Syzygium aromaticum* (clove)	Eugenol	*Staphylococcus aureus*, and *Candida albicans*	(339)
*Mentha piperita* (peppermint)	Menthol, and menthone	*Escherichia coli*, and *Staphylococcus aureus*	(340)
*Piper nigrum* (black pepper)	Piperine	*Escherichia coli*, and *Staphylococcus aureus*	(341)

#### 5.2.1 Phenolic compounds

These secondary metabolites comprise phenolic and hydroxyl functional groups linked to an aromatic ring known as phenol. Phenol itself is made up of chemically varied groupings. Gautam et al. (178) assert that phenolic chemicals serve multiple protective functions in nature, including facilitating healthy plant development and reproduction, enhancing seed germination prior to harvest, and providing protection against diseases and predators (178). Four distinct categories of polyphenols comprise phenolic acids, flavonoids, tannins, and stilbenes (179). Alkyl esters of hydroxybenzoic acid parabens are widely utilized as antimicrobials and preservatives in pharmaceuticals, cosmetics, food, and drinks (179, 180).

Flavonoids are a significant subset of polyphenolic compounds, encompassing over 9,000 recognized natural molecules. Various kinds of flavonoids have been ascribed with antiallergic, antidiabetic, anti-inflammatory, antiviral, antibacterial, antifungal, antiproliferative, anticarcinogenic, hepatoprotective, and antioxidant properties (181). Moreover, numerous investigations have demonstrated the effectiveness of these compounds in addressing MDR bacteria of both Gram-negative and Gram-positive types (182). In reaction to microbial infection, plants synthesize secondary metabolites; thus, the extensive *in vitro* data on their broad-spectrum antibacterial efficacy aligns effectively (182). Tannins are extensively distributed in various plant parts, and it has been suggested that they serve as a natural defense against microbial invasion. Tannins exhibit antibacterial effects against fungi, yeasts, bacteria, and viruses. Tannic acid and propyl gallate exhibit inhibitory effect against food-borne bacteria, aquatic bacteria, and microorganisms that affect flavor (182, 183).

Aristri et al. (183) reported that tannins had antibacterial properties, which appear to be linked to the cleavage of the ester linkage between gallic acid and polyols. The disruption of DNA synthesis, the inactivation of cellular enzymes, the alteration of membrane fluidity, and the inhibition of protein synthesis have all been associated with the antimicrobial effects of various phenolic compounds (183).

#### 5.2.2 Terpenoids

Terpenoids are the predominant secondary metabolites, originating metabolically from acetyl-CoA or glycolytic intermediates. Plants produce several terpenoids as secondary metabolites, which are thought to provide protection against insect and mammalian herbivores (184). The molecular structure common to these metabolites is (C_10_H_16_). The antibacterial properties of several terpenes against bacteria, fungi, viruses, and protozoa have been recorded (184). Oxygenated terpenoids with varied structures exhibit antibacterial activity against numerous bacteria and fungi, especially those with alcohol functional groups. These molecules demonstrate superior action relative to aldehydes and ketones. While the precise mechanism of action of these terpenoids remains unclear, it is hypothesized that they may compromise cell membranes (185).

Farha et al. (186) investigated the efficacy of (+)-nootkatone against biofilms of MDR *S. aureus* and analyzed its hypothesized molecular mechanism. (+)-Nootkatone shown bactericidal efficacy against *S. aureus* strains SJTUF 20758 and ATCC 25343 (186). Light microscopy and confocal laser scanning microscopy demonstrated that (+)-nootkatone significantly diminished biofilm thickness. The bacterial growth curve indicated that the antibacterial efficacy of (+)-nootkatone was dosage-dependent, with dosages below the MIC failing to impede the proliferation of free-floating bacterial cells (186).

Additionally, (+)-nootkatone decreased the motility of *S. aureus*. At a concentration of 200 grams/ml, (+)-nootkatone decreased biofilm by 50% and eradicated 80% of bacterial cells. The transcriptional analysis demonstrated that (+)-nootkatone inhibited the expression of genes associated with biofilm development, including Sara, icaA, Agra, RNAIII, and spa (186). Moreover, the MTT assay indicated that (+)-nootkatone displayed no toxicity to human foreskin fibroblast (HFF) cells. Consequently, (+)-nootkatone is a potentially safe phytochemical for application in the food industry (186).

#### 5.2.3 Alkaloids

Nitrogen constitutes a component of alkaloids, which are secondary metabolites prevalent in numerous plants. These metabolites comprising organic acids, exhibit hemolytic activity, and are toxic to bacteria (187). For 4000 years, people have cohabited with and employed alkaloid-rich plants. In the early 1800s, the therapeutically active constituents of this medication class were found (187). The analgesic and narcotic effects of the morphine component obtained from *Papaver somniferum* (opium poppy) have been utilized. After several years, researchers identified substances like strychnine, emetine, brucine, piperine, caffeine, quinine, cinchonine, and colchicine. To far, over 10,000 alkaloids have been recorded (188).

Alkaloids exhibit significant biological activity owing to their capacity to establish hydrogen bonds with enzymes, receptors, and proteins. This capability is enabled by a nitrogen atom that absorbs protons and one or more amine hydrogen atoms that contribute protons (188). Alkaloids have gained significance in the treatment of infectious diseases exhibiting MDR due to their antibacterial capabilities. Consequently, scientists focus on these intriguing secondary plant metabolites (188, 189). To obtain pure alkaloids, it is essential to devise alternate extraction procedures, as their natural sources typically yield very minimal quantities (189).

#### 5.2.4 Glycosides

These secondary metabolites comprise glucose or an alternative sugar combined with non-sugar molecules, such as terpenes or phenolic compounds. Cyanogenic glycosides, saponins, solanines, and mustard oil glycosides are the toxic glycosides implicated in plant poisonings (190). Nonetheless, several glycosides, including cardiac glycosides, have been empirically documented to offer therapeutic benefits, especially in the management of heart disease and cancer (191).

MRSA and MDR *Acinetobacter baumannii* (MDRAB) pose substantial challenges owing to their ability to acquire resistance to commonly utilized antibiotics. This resistance enables them to induce chronic infections and impede the healing process (192). El-Shiekh et al. (192) investigated the efficacy of *Caralluma quadrangular* extracts (MeOH, CH_2_Cl_2_, and n-butanol) against MDR MRSA USA300 and *A. baumannii* AB5057. The MeOH extract and both fractions of *C. quadrangular* demonstrated a significant decrease in biofilm formation *in vitro*. All doses (0.625, 0.313, and 0.156 mg/ml) eliminated pre-existing MRSA and MDRAB biofilms (192).

*C. quadrangular* extracts significantly decreased bacterial loads in MRSA-infected dermal lesions in murine models (192). Four pregnane glycosides and one flavone glycoside were isolated from the bioactive n-butanol fraction. The biofilm inhibition and detachment properties of the isolated compounds (Rus A–E) were assessed. Rus C demonstrated the greatest activity level among the compounds, with an IC_50_ value of 0.139 mmole, whilst Rus E exhibited the lowest activity level, with an IC_50_ value of 0.818 mmole (192). The results demonstrated that extracts from *C. quadrangular* or its constituents could diminish biofilm adherence and the pathogenicity of MRSA and MDRAB. Furthermore, they may serve as a topical antibacterial treatment for MRSA skin infections (192).

Strophanthin, a steroidal glycoside derived from *Moringa oleifera* seeds, surpasses alum in its efficacy to aggregate and precipitate inorganic and organic materials in wastewater (193). Furthermore, it has been demonstrated to decrease microbial load by 55% and coliform burden by 65% (193, 194). The exact mechanism of cardiac glycosides is not fully understood; however, it is posited that they inhibit Na^+^/K^+^-ATPase by binding to its receptor, resulting in elevated intracellular sodium ion concentrations. Consequently, the regulation of Na^+^/Ca^2+^ transport across the cell membrane is modified, leading to advantageous inotropic effects (195).

#### 5.2.5 Lectins and polypeptides

Lectins are proteins that have a great attraction for carbohydrates, namely the glycosidic bonds found in the seeds and tubers of various plants, such as wheat, potatoes, and beans (196). More significant lectin molecules, such as MAP30 from bitter melon and GAP31 from *Gelonium multiflorum*, have demonstrated the ability to inhibit viral replication (HIV, CMV) by interfering with viral interactions with crucial host cell components (197). Peptides exhibit a cationic charge and disulfide bonds, allowing them to inhibit microbial development. Thionins, often found in barley and wheat, inhibit the proliferation of yeasts and both Gram-negative and Gram-positive bacteria (198).

Pandey and Srivastava (199) demonstrated that the sugar beet thionins AX1 and AX2 had antifungal properties but lacked antibacterial activity. In a study by Bilal et al. (200), fabatin was found to have effects comparable to g-thionins, diminishing antibacterial efficacy against *E. coli*, *P. aeruginosa*, and *Enterococcus hirae*. The sulfur-containing secondary metabolites may enhance their activity by promoting the development of ion channels in the cell membrane (196).

## 6 Biological activities of medicinal plants

The benefits of medicinal plants, including their antibacterial, antioxidant, and anticancer characteristics, are illustrated in **Table 2**.

**Table 2:** Benefits of medicinal plants and their antibacterial, antioxidant, and anticancer properties.

Medicinal plants	Geographical origin	*In vitro* and/or *in vivo* biological activities	References
*Hypericum perforatum* (St. John’s wort)	Europe, Asia, and North Africa	Demonstrated *in vitro* antibacterial activity against *Pseudomonas aeruginosa*, *Escherichia coli*, *Enterococcus faecalis*, *Klebsiella oxytoca*, *Klebsiella pneumoniae*, and *Staphylococcus aureus*	(342)
*Anethum graveolens* (dill)	Mediterranean, Europe, and Asia	Demonstrated *in vitro* antimicrobial activity against *Candida albicans*, and *Staphylococcus aureus*	(343)
*Salvia officinalis* (sage)	Mediterranean	Demonstrated *in vitro* antimicrobial properties against *Bacillus cereus*, evaluated via thin layer chromatography (TLC) investigations	(344)
*Pyrus salicifolia* (willow-leaved pear)	Asia	Demonstrated *in vitro* antimicrobial activities against *Bacillus subtilis, Bacillus pumalis, Candida albicans, Escherichia coli, Pseudomonas aeruginosa*, and *Staphylococcus aureus*	(345)
*Dioscorea bulbifera* (air potato)	Tropical Africa, and Asia	Demonstrated *in vitro* antimicrobial activities against *Bacillus subtilis, Bacillus pumalis, Candida albicans, Escherichia coli, Pseudomonas aeruginosa*, and *Staphylococcus aureus*	(346)
*Artemisia dracunculus* (tarragon)	Eurasia	Demonstrated *in vitro* antimicrobial activities against *Candida albicans*, *Xanthomonas maltophilia*, and *Proteus mirabilis*	(347)
*Acroptilon repens* (Russian knapweed)	Central Asia	Demonstrated *in vitro* antibacterial activity against *Staphylococcus saprophyticus*, and *Staphylococcus epidermidis*	(348)
*Cuminum cyminum* (cumin), and *Carum carvi* (caraway)	Middle East, and Europe	Demonstrated *in vitro* antibacterial activity via diffusion method against various Gram-positive and Gram-negative bacteria, including the genera *Clavibacter, Curtobacterium, Rhodococcus, Erwinia, Xanthomonas, Ralstonia*, and *Agrobacterium*	(349)
*Zingiber officinale* (ginger)	Southeast Asia	Demonstrated *in vivo* anti-tumor growth by penetrating the tumor site and presumably increasing the permeability and retention (EPR) impact	(350)
*Juncus effusus* (soft rush)	Worldwide	Showed *in vivo* hepatoprotective activity (reduction of liver damage markers in blood)	(351)
*Paeonia lactiflora* (Chinese peony)	Asia	Showed *in vivo* hepatoprotective impact (enhanced free radical scavenging, inhibition of lipid peroxidation, control of bilirubin and cholic acid metabolism)	(352)
*Silene wallichiana*, and *Silene viridiflora* (campion)	Himalayas (Nepal, India, and Bhutan)	Displayed *in vitro* antibacterial, moderate radical scavenging, and antioxidant properties	(353)
*Lonicera japonica* (Japanese honeysuckle)	East Asia (China, Japan, and Korea)	Showed *in vitro* and *in vivo* activity against *Staphylococcus aureus*, and *Streptococcus pyogenes*. Showed anti-inflammatory effect by reducing LPS-induced fever, hypothermia, and inflammation (decreased TNF-α, IL-1β, IL-6 serum concentrations)	(354)
*Citrus aurantium* (bitter orange)	Southeast Asia	Showed *in vivo* anti-gouty effect by inhibiting xanthine oxidase activities (serum and liver) and reducing hyperuricemia	(355)
*Pueraria montana* var. lobata (Kudzu)	East Asia (China, and Japan)	Showed *in vivo* anti-gouty effect by reducing swelling, alleviating pathological damage to gouty arthritis, and lowering blood uric acid (inhibited xanthine oxidase activity)	(356)
*Thymus seravschanicus* (creeping tyme)	Central Asia	Showed *in vitro* antioxidant and *in vivo* antibacterial activity. It also inhibited *Helicobacter pylori* growth	(357)
*Arctium lappa* (burdock)	Europe, and Asia	Showed *in vitro* synergistic effect by increasing common antibiotics’ *in vitro* antimicrobial efficacy (Gram-positive and Gram-negative bacteria)	(358)
*Arctium lappa* (greater burdock)	East Asia (China)	Showed *in vitro* anti-obesity, antimicrobial, hypocholesterolemic, and hypolipidemic activity	(359)
*Phellodendron chinense* (Chinese corktree)	East Asia (China)	Showed *in vivo* anti-psoriasis effect via regulating polarization	(360)
*Origanum tyttanthum* (oregano)	Mediterranean	Used to treat *in vivo* skin wounds and ulcers (stimulated healing process)	(361)
*Aconitum* spp. (aconite), *Agrimonia eupatoria* (common agrimony)	Europe, and Asia	Showed *in vitro* antiviral and inhibitory activity against Hepatitis B virus	(362)
*Akebia trifoliata* (chocolate vine)	East Asia (China, Korea, and Japan)	Showed *in vitro* and *in vivo* anti-inflammatory activity (chronic inflammations), ameliorates inflammation (NF-κB/MAPK signaling pathways), and modifies gut microbiota	(363)
*Origanum vulgare* sp. *vulgare* (oregano)	Mediterranean, Europe, and Asia	Showed *in vitro* antibacterial activity against various Gram-negative bacteria (*Aeromonas hydrophila*, *Citrobacter* sp., *Enterobacter aerogenes*, *Escherichia coli*, *Flavobacterium* sp., *Klebsiella* spp., *Proteus mirabilis*, *Pseudomonas aeruginosa*, *Salmonella* spp., *Serratia marcescens*, and *Shigella dysenteriae*)	(364)
*Plumbago zeylanica* (Ceylon leadwort)	Tropical Africa, and Asia	Showed *in vitro* anti-inflammatory activity (regulated bacterial virulence factors, and biofilms)	(365)
*Crinum glaucum* (seashore lily)	Africa	Showed *in vitro* anti-inflammatory and antimicrobial activity against ten human pathogenic bacteria	(366)
*Zingiber officinale* (ginger), *Thymus kotschyanus*	Southeast Asia, and Iran	Showed *in vitro* antimicrobial effect; suppressed *Staphylococcus aureus* and *Escherichia coli* growth	(367)
*Zingiber officinale* (ginger), *Allium sativum* (garlic)	Southeast Asia, and Central Asia	Inhibited *Staphylococcus aureus* growth *in vitro*	(368)
*Tribulus terrestris* (bull’s head), and *Soymida febrifuga* (Indian redwood)	Worldwide, and India	Inhibited *Escherichia coli*, *Enterococcus faecalis*, *Klebsiella oxytoca*, and *Staphylococcus aureus in vitro*	(369)

### 6.1 Antioxidant and anticancer activities

Medicinal herbs include many active compounds, whether fresh, dried, or crushed. These constitute the essential principles of phytotherapy. Conversely, the majority of conventional pharmaceutical drugs, including those with components derived from medicinal plants, function as a chemical marker with a singular active ingredient (201). Cinnamon exemplifies these two processes effectively. Historically, infectious diseases were treated with an infusion of cinnamon bark. The primary secondary metabolite of the bark, cinnamaldehyde, demonstrated efficacy as an antibacterial agent and may serve as a traditional remedy comparable to synthetic antimicrobials (201).

About 67% of anti-cancer pharmaceuticals comprise chemicals derived from or primarily produced from flora or fauna (201). Many are employed in the treatment of cancer, particularly as chemotherapeutics. The FDA has included taxol (Paclitaxel), a phenolic chemical sourced from the bark of *Taxus brevifolia*, in its roster of authorized cancer medications (201). Moreover, several extracts are available as commercially sold goods. Xuancheng Baicao Plants Industry and Trade Ltd., situated in China, offers powdered *Lonicera* (honeysuckles) extract containing 25% chlorogenic acid and *Ganoderma* extract with a 30% polysaccharide content (202).

The efficacy of solvents employed to extract active compounds from medicinal plants varies. Moreover, preparations of medicinal plants and essential oils exhibit significant antioxidant and antiviral activities. Clove extract exhibits superior antioxidant action compared to vitamin C against 2,2-diphenyl-1-picrylhydrazyl (DPPH), 2,2′-casino-bis (3-ethylbenzothiazole-6-sulfonic acid) (ABTS), and superoxide radicals. Moreover, clove extract and essential oil have antiviral properties (202). The chemical composition of medicinal plants, especially the occurrence of phenolic compounds, is linked to their notable antioxidant and antibacterial properties in extracts and essential oils (113).

### 6.2 Antimicrobial activity of medicinal plants against MDR microorganisms

Before the discovery of microorganisms and their functions, humanity has traditionally recognized plants as a source of medical efficacy (202, 203). Medicinal plants are effective against various viruses, including herpes, adenovirus, poliovirus, and coxsackievirus, and possess anticancer and anti-inflammatory properties (203). In light of the increasing use of herbal therapy as an alternative to traditional antibiotics and the proliferation of antibiotic-resistant bacteria, numerous studies have been conducted on the antibacterial properties of medicinal herbs (204, 205). These medicinal plants are acknowledged to possess significant untapped bioactive chemical potential (205–208).

Numerous therapeutic plant extracts possess secondary metabolites exhibiting significant efficacy against MDR microorganisms. Panda et al. (209) demonstrated a range of ethnobotanical plants possessing MDR characteristics. The acetone extract of the leaves of *Smilax zeylanica* and *Syzygium praecox*, known for their efficacy in treating ulcers and skin infections, showed antibacterial activity against MRSA by compromising cellular membranes via ionic and electrostatic interactions (209). Moreover, the ethanol extract of long-leaf varnish tree fruit exhibits MDR activity against *E. faecium*, *S. aureus*, *K. pneumoniae*, *A. baumannii*, *P. aeruginosa*, and *Enterobacter* species via ionic interactions between terpenoids and steroid compounds in the extract and the bacterial cell membrane (209).

The ethanol extract of climbing acacia leaves is utilized ethnobotanically to alleviate dysentery and diarrhea, as well as to reduce cholesterol levels, whilst the acetone extract of java cedar leaves is employed to address skin ailments. The antimicrobial activity of both plants against *K. pneumoniae*, *A. baumannii*, *P. aeruginosa*, and *Enterobacter* species is attributed to electrostatic interactions between positively charged secondary metabolites and negatively charged bacterial cell membranes. The amphipathic properties of Ceylon olive blossom aqua extract, Buddha coconut bark ethanol extract, and charcoal tree leaves acetone extract facilitate concurrent interaction with MDR bacterial cells, including *K. pneumoniae*, *A. baumannii*, *P. aeruginosa*, and *Enterobacter* species (209). Furthermore, the extracts employed in traditional medicine for the treatment of cardiovascular diseases, dermatological infections, and wound infections (209).

Richwagen et al. (210) indicated that the ethanol extract of lavender woody stems may alleviate pain through ionic interactions between the cell membranes of MDR *S. aureus*, *A. baumannii*, and *P. aeruginosa* and the flavonoid extract. The methanol extract of coastal golden leaf and bark is traditionally recognized for its efficacy in addressing digestive disorders, ocular issues, and infertility, exhibiting hepatoprotective, antioxidant, anticancer, antiviral, and antibacterial attributes (210). The amphipathic characteristics of the plant contribute to its antibacterial efficacy against MDR *S. aureus*, *K. pneumoniae*, *P. aeruginosa*, *Enterobacter* species, and MRSA (210).

Pallah et al. (211) demonstrated a range of plants exhibiting MDR activities. Cherry plum, plum, red currant, and methanol extracts exhibited antioxidant, anti-inflammatory, and antibacterial activities against MDR species, including *S. aureus*, *K. pneumoniae*, and *P. aeruginosa*, through electrostatic interactions (211). The Jostaberry methanol extract demonstrated inhibitory zones against *S. aureus* and *P. aeruginosa* using the same method (211). Additionally, methanol extract of sweet cherry fruit is conventionally employed to alleviate osteoarthritis and address cardiovascular illnesses. Sweet cherry exhibited antibacterial action against MDR organisms *S. aureus*, *K. pneumoniae*, and *P. aeruginosa* owing to its amphipathic characteristics (211).

The complete plant ethanol extract of Maidenhair fern and Chirayita is effective against *S. aureus*, and *E. faecium*. The extract of maidenhair fern possesses astringent, demulcent, expectorant, and diuretic characteristics, successfully addressing urinary tract infections (212). Chirayita extract is utilized for inflammation, hepatitis, digestive disorders, chronic fever, malaria, dermatological conditions, and bronchial infections. Both extracts combat infections via amphipathicity (212). The extract from aerial portions addresses pruritus, viral inflammatory dermatoses, malaria, and typhoid, specifically targeting *E. faecium* and *P. aeruginosa* through electrostatic interactions. Timber fruit extract addresses cancer, gastrointestinal disorders such as cholera and dysentery, and oral infections including toothaches and mouth ulcers. It additionally targets *E. faecium* and *S. aureus* through electrostatic interactions (212).

Shobha et al. (213) investigated the antibacterial properties of an ethanol extract derived from cashew leaves. The herb is conventionally utilized for venereal disorders, gastrointestinal issues, dermatological conditions, stomatitis, bronchitis, psoriasis, dental pain, and periodontal infections. The extract comprises hydrophobic and hydrophilic residues, hence demonstrating MDR antibacterial action against *S. aureus*, *K. pneumoniae*, and *P. aeruginosa* (213).

Chandrasekharan et al. (214) investigated the therapeutic applications and mechanisms of several medicinal plant extracts against MDR microorganisms. The pipli root aqueous extract and the ethanol extract of Bermuda grass leaves function via electrostatic interactions. Pipli root extract is recognized for its antioxidant and anti-inflammatory activities, especially against *E. faecium* and *A. baumannii* (214). The ethanol extract of Bermuda grass leaves serves as a laxative, tonic for the brain and heart, and a cooling agent to eliminate *S. aureus* (214). The hexane extract of lemongrass modulates hypertension, epilepsy, and gastrointestinal and central nervous system problems, specifically targeting *P. aeruginosa* and *E. faecium* via ionic interactions (214). *Aloe* extract facilitates wound healing, mitigates inflammation, and addresses burn-related skin injuries by rebuilding compromised skin. It targets *P. aeruginosa* and *Enterococcus casseliflavus* through amphipathicity, encompassing hydrophobic and hydrophilic residues (214).

Nayim et al. (215) indicate that certain plant extracts exhibit significant antibacterial efficacy against MDR bacteria due to ionic interactions. Methanol extracts from cocoa seeds, bush butter tree, and okra leaves suppress MDR *K. pneumoniae*, but sweet potato leaf extract targets the same bacteria through amphipathicity (215). These plants possess traditional therapeutic applications: cocoa seeds for disorders such as dermal injuries and diarrhea, bush butter tree for tonsillitis, okra leaves for infections, and sweet potato leaves for conditions including diabetes and inflammation (215).

Likewise, extracts from neem tree bark and pomelo leaves demonstrate efficacy against MDR *Enterobacter* species via electrostatic interactions (215). Neem bark is typically utilized for dermatological conditions and febrile illnesses, whereas pomelo leaves are employed for neurological problems and hemorrhagic conditions (215). Winter squash bean extract, utilized in traditional therapy for renal and gastrointestinal ailments, also addresses MDR *Enterobacter* and *K. pneumoniae* via amphipathicity (215). Ultimately, common bean leaf extract suppresses MDR *E. casseliflavus* and is commonly utilized for diabetes and weight management (215).

Subramani et al. (216) identified multiple plants demonstrating MDR activities. The methanol extract of *Lantana* leaves yields secondary compounds that mitigate inflammation, combat malaria, alleviate spasms, suppress tumor proliferation, avert ulcer development, and specifically target *Enterobacter* species and *P. aeruginosa* through electrostatic interactions (216). The alcoholic extract of myrtle leaves showed efficacy against *S. aureus* and *P. aeruginosa* via electrostatic interactions (216). It addresses cancer, inflammation, diabetes, ulcers, hypertension, diarrhea, and rheumatism. Extracts of Palash (*Butea monosperma*) leaves (ethanol, hexane, and water) enhanced diuresis and menstrual flow while suppressing *Enterobacter* species through ionic interactions (216). The ethanol extract of Burflower (*Neolamarckia cadamba*) tree leaves and bark alleviated fever, uterine ailments, skin problems, and inflammation, while also functioning as a febrifuge, antidiarrheal, antihyperglycemic, and antibacterial agent against *A. baumannii* and *P. aeruginosa*, attributed to their amphipathic characteristics (216).

Masoumian and Zandi (217) demonstrated the therapeutic efficacy of various plant extracts against MDR species. The extract of cinnamon bark water is efficacious in managing diabetes, reducing oxidative stress, and mitigating inflammation. This extract shows efficacy against *S. aureus* and *P. aeruginosa* due to ionic interactions (217). Similarly, medicinal *Aloe* leaf water extracts precisely target *S. aureus* and *P. aeruginosa*, facilitating the treatment of digestive issues and burns via ionic interactions. The aqueous extract of mint leaves has shown efficacy against acid reflux, irritable bowel syndrome, and the common cold (217). It demonstrated efficacy against *S. aureus* and *P. aeruginosa* owing to its amphipathic metabolites, enabling the extract to engage concurrently with hydrophobic and hydrophilic residues (217). The aqueous extract of henna leaves exhibited antibacterial activity against *S. aureus* and *P. aeruginosa* via electrostatic interactions. It proficiently addressed burns, wounds, and cutaneous infections while enhancing hair vitality (217).

Likewise, the chloroform extract of snake flower leaves effectively addressed skin and wound infections due to the hydrophobic properties of the plant extract (218). Ghuman et al. (219) showed that chloroform extract from bitter *Aloe* leaves can address skin problems by targeting *S. aureus*, *P. aeruginosa*, and *A. baumannii*. The extract’s efficacy stemmed from its capacity to adhere to the hydrophobic groups located in the bacterial membrane (219). Furthermore, the ethanol extract of hrse mint (*Mentha longifolia*) aerial parts significantly mitigated throat and oral discomfort, addressing sore throats and targeting *Mycobacterium tuberculosis* through its amphipathic characteristics (219).

Agarwal et al. (220) indicated that the ethanol extract of *Phyllanthus emblica* (Indian gooseberry) aerial parts could effectively target *M. tuberculosis* and MRSA via electrostatic interactions (220). Moreover, the dichloromethane extract of tree *Aloe* leaves demonstrated effectiveness in addressing skin, gastrointestinal, and respiratory ailments, countering *S. aureus* and *P. aeruginosa* owing to their hydrophobic properties (220). Bacha et al. (221) examined the antibacterial efficacy of methanol extract from cardamom (*Elettaria cardamomum* ***)*** fruit against *S. aureus*. The mechanism of action entails ionic and electrostatic interactions that result in the deposition of the plant extract on the bacterial cell membrane, inducing instability and disintegration of the cellular membrane (221).

Additionally, Arullappan et al. (222) demonstrated that the leaf extract of *Clinacanthus nutans* had a notable antifungal activity against *C. albicans* in a laboratory environment, with MIC above 1 mg/ml. Numerous research, including those by Ortega‐Ramirez et al. (223), Ma et al. (224), and Shanmugam et al. (225), have elucidated the antibacterial characteristics of *Allium cepa* (onion). Zhou et al. (226) demonstrated that a gel including *Durio zibethinus* polysaccharide at a dose of 100 mg/mL efficiently suppresses the proliferation of oral infections. Diris et al. (227), and Apridamayanti et al. (228) reported the antibacterial properties of Malabar melastome (*Melastoma malabathricum*). The inhibitory activity of this plant extract may be ascribed to its phenolic constituents, such as flavonols and ellagic acid (229).

Under laboratory conditions, the fruit extract of *Cucurbita maxima* (Buttercup squash) has demonstrated antibacterial efficacy against *S. aureus*, *Bacillus subtilis*, and *Micrococcus luteus* (230). The constituent of this fruit, which comprises caffeic acid and p-coumaric acid, possesses antibacterial properties (230). Numerous research studies have demonstrated the antibacterial efficacy of *Zingiber cassumunar* (cassumunar ginger) essential oil against dermatophytes (231, 232). Research conducted by (33) demonstrated that the traditional herbal preparation known as “Loloh,” commonly prepared as a decoction on Bali Island, is effective in treating several ailments caused by pathogens, including diarrhea and dysentery. Numerous investigations have demonstrated the antibacterial efficacy of *Andrographis paniculata* (green chiretta) under *in vitro* conditions against Gram-positive and Gram-negative bacteria (233–235).

Andrographolide, the principal active constituent from the stem and leaves of *Andrographis paniculata*, has demonstrated significant efficacy as an anti-diarrheal agent in clinical trials and is accountable for the plant’s antibacterial properties (236, 237). The antibacterial properties of *Blumea balsamifera* (ngai camphor), *Jatropha curcas* (barbados nut), and *Lablab purpureus* (hyacinth bean) have been recorded in the literature reviewed by Priya and Karthika (238) and Rampadarath et al. (239).

Women in Minahasa, North Sulawesi, Indonesia, frequently utilize Baker, a traditional herbal steam bath, for thermotherapy, aromatherapy, and postpartum recuperation. Alexa et al. (240) indicated that 31 species utilized in “Baker” produce essential oils, including *Citrus hystrix*, *Curcuma xanthorrhiza*, *Cymbopogon citratus*, *Cymbopogon nardus*, *Myristica fragrans*, and *Syzygium aromaticum*. The volatile oil constituents (eugenol, β-pinene, sabinene, and zingiberene) linked to the medicinal benefits of these plants have been identified (240).

Eugenol derived from *Syzygium aromaticum* (clove) exhibited antibacterial properties against *S. aureus* ATCC 29213 and MRSA clinical isolates. Furthermore, it impeded the production of biofilms, hence diminishing the colonization of *S. aureus in vivo* (241). It efficiently eradicates existing biofilms, diminishes the expression of genes linked to biofilms and enterotoxins, disrupts the cell membrane, and results in the release of cellular contents (241). Moreover, eugenol has demonstrated antifungal capabilities and the capacity to inhibit oral microorganisms associated with periodontal disease and dental caries (242–244). Eugenol is readily accessible and utilized as a counterirritant, topical antiseptic, and in dentistry, frequently coupled with zinc oxide for analgesia and root canal obturation (244, 245).

## 7 Antimicrobial mechanism of medicinal plants against MDR

**Figure 5:**
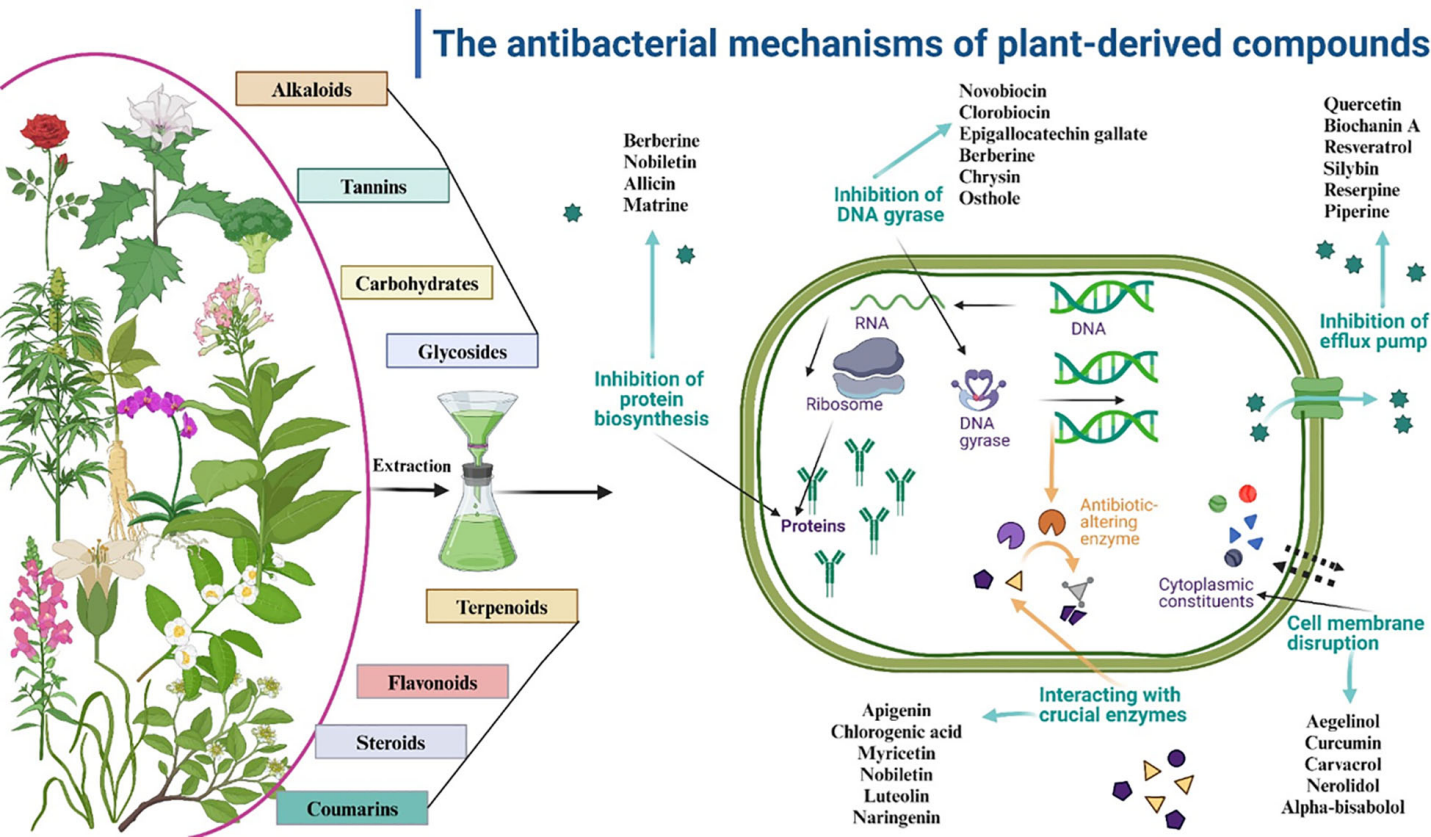
Antibacterial mechanism of medicinal plants against multi-drug resistance (MDR) as natural alternatives to antibiotics.

Medicinal plants have gained significant attention for their potential to combat MDR microorganisms due to their diverse biochemical and molecular mechanisms (173, 182, 208). The action mechanism of certain medicinal herbs against MDR microorganisms is illustrated in **Table 3**. Furthermore, **Figure 5** illustrates the antibacterial mechanism they employ to combat MDR. These mechanisms often differ from conventional antibiotics, making them promising alternatives in the fight against AMR (**Figure 5**).

**Table 3:** Mechanism of action of some medicinal plants against multi-drug resistant (MDR) microorganisms.

Medicinal plant extracts	Applications	MDR microorganisms	Mechanism of action	References
Acetone extract from *Smilax zeylanica* leaves	Ulcers treatment	Methicillin-resistant *Staphylococcus aureus* (MRSA)	Cellular membrane instability and disintegration are brought on by the plant extract’s ionic/electrostatic interactions and deposition on the bilayer surface	(209)
Acetone extract from *Syzygium praecox* leaves	Skin infection treatment	Methicillin-resistant *Staphylococcus aureus* (MRSA)	The plant extract and cell membrane interact via ionic/electrostatic forces	(209)
Aqueous extract from Ceylon olive flower	Exhibited diuretic and cardiovascular stimulant characteristics	*Enterococcus faecium*, *Staphylococcus aureus*, *Klebsiella pneumoniae*, *Acinetobacter baumannii*, *Pseudomonas aeruginosa*, and *Enterobacter* species	Amphipathicity refers to the presence of both hydrophobic and hydrophilic residues, and both processes may be operating simultaneously	(209)
Ethanol extract from climbing acacia leaves	Treating dysentery, diarrhea and lowering cholesterol levels	*Enterococcus faecium*, *Staphylococcus aureus*, *Klebsiella pneumoniae*, *Acinetobacter baumannii*, *Pseudomonas aeruginosa*, and *Enterobacter* species	Electrostatic interactions	(209)
Ethanol extract from long-leaf varnish tree fruit	Skin diseases treatment	*Enterococcus faecium*, *Staphylococcus aureus*, *Klebsiella pneumoniae*, *Acinetobacter baumannii*, *Pseudomonas aeruginosa*, and *Enterobacter* species	Ionic interactions	(209)
Ethanol extract from orange jessamine leaves	Cardiovascular disorders treatment	Methicillin-resistant *Staphylococcus aureus* (MRSA)	Hydrophobicity of the extract	(209)
Ethanol extract from Buddha coconut bark	Skin diseases treatment	*Enterococcus faecium*, *Staphylococcus aureus*, *Klebsiella pneumoniae*, *Acinetobacter baumannii*, *Pseudomonas aeruginosa*, and *Enterobacter* species	Amphipathicity refers to the presence of hydrophobic and hydrophilic residues, which may operate simultaneously	(209)
Acetone extract from cinnamon leaves	Throat and skin infection treatment	Methicillin-resistant *Staphylococcus aureus* (MRSA)	The hydrophobicity of the plant extract might influence the reaction’s outcome since it binds to the hydrophobic groups present in the bacterial membrane	(209)
Acetone extract from charcoal tree leaves	Boils, sore throat, and wound infections treatment	*Enterococcus faecium*, *Staphylococcus aureus*, *Klebsiella pneumoniae*, *Acinetobacter baumannii*, *Pseudomonas aeruginosa*, and *Enterobacter* species	Amphipathicity denotes the coexistence of hydrophobic and hydrophilic residues, with both processes potentially occurring concurrently	(209)
Acetone extract from Java cedar leaves	Skin diseases treatment	*Enterococcus faecium*, *Staphylococcus aureus*, *Klebsiella pneumoniae*, *Acinetobacter baumannii*, *Pseudomonas aeruginosa*, and *Enterobacter* species	Electrostatic interactions	(209)
Ethanol extract from lavender woody stems	A pain reliever treatment	*Staphylococcus aureus*, *Acinetobacter baumannii*, and *Pseudomonas aeruginosa*	Ionic interactions	(210)
Methanol extract from coastal golden leaf and bark	Amebic dysentery, cough, diarrhea, stomach ulcer, eye problems, infertility, and tapeworm treatment. Showed antimicrobial, hepatoprotective, antioxidant, anticancer, and antiviral properties	*Staphylococcus aureus*, *Klebsiella pneumoniae*, *Pseudomonas aeruginosa*, MRSA, and *Enterobacter* species	Amphipathicity is defined as the existence of both hydrophobic and hydrophilic residues, which can occur concurrently	(370)
Methanol extract from cherry plum fruit	Considerable properties, including astringent, antioxidant, sudorific, antipyretic, laxative, and diuretic	*Klebsiella pneumoniae*, *Staphylococcus aureus*, and *Pseudomonas aeruginosa*	Electrostatic interactions	(211)
Methanol extract from jostaberry	Improved Immunity. Act as antiaging characters. Cardiovascular diseases treatment	*Staphylococcus aureus*, and *Pseudomonas aeruginosa*	Ionic interactions	(211)
Methanol extract from sweet cherry fruit	Cancer, osteoarthritis, and cardiovascular disease treatment	*Staphylococcus aureus*, *Klebsiella pneumoniae*, and *Pseudomonas aeruginosa*	Amphipathicity is defined as the existence of both hydrophobic and hydrophilic residues, which can occur concurrently.	(211)
Methanol extract from plum fruit	Antioxidant, anti-inflammatory, and memory-enhancing properties	*Staphylococcus aureus*, *Klebsiella pneumoniae*, and *Pseudomonas aeruginosa*	Electrostatic interactions	(211)
Methanol extract from red currant fruit	Scurvy and alleviate constipation, digestive and urinary problems treatment	*Staphylococcus aureus*, *Klebsiella pneumoniae*, and *Pseudomonas aeruginosa*	Ionic interactions	(211)
Ethanol extract from *Adiantum* (maidenhair fern) whole plant	Urinary tract infections treatment. An astringent, demulcent, expectorant, and diuretic	*Enterococcus faecium*, and *Staphylococcus aureus*	Amphipathicity denotes the coexistence of hydrophobic and hydrophilic residues, with both processes potentially occurring concurrently	(212)
Ethanol extract from aerial parts of Bichoo bel	Pruritus treatment. Viral inflammatory skin conditions. Malaria, and typhoid treatment	*Enterococcus faecium*, and *Pseudomonas aeruginosa*	Electrostatic interactions	(212)
Ethanol extract from Bichoo bel fruit	Sore throat treatment. Wound healing. Skin disorders treatment	*Enterococcus faecium*, *Staphylococcus aureus*, *Klebsiella pneumoniae*, and *Acinetobacter baumannii*	Ionic interactions	(212)
Ethanol extract from *Swertia chirayita* (Chirata) whole plant	Inflammation, hepatitis, digestive disorders, chronic fever, malaria, skin disease, and bronchial infections treatment	*Staphylococcus aureus*	Amphipathicity denotes the coexistence of hydrophobic and hydrophilic residues, with both processes potentially occurring concurrently	(212)
Ethanol extract from timber fruit	Cancer, gastrointestinal conditions such as cholera and dysentery, tooth infections, and mouth ulcers treatment	*Enterococcus faecium*, and *Staphylococcus aureus*	Electrostatic interactions	(212)
Ethanol extract from *Berberis* *lycium* (Indian lyceum) root	Dental infections, toothaches, and earaches treatment. Utilized historically to treat diarrhea, cholera, and piles	*Enterococcus faecium*	Ionic interactions	(212)
Ethanol extract from cashew leaves	Treatment for venereal problems, stomach problems, skin diseases, stomatitis, bronchitis, psoriasis, toothaches, and gum infections	*Staphylococcus aureus*, *Klebsiella pneumoniae*, and *Pseudomonas aeruginosa*	Amphipathicity is defined as the existence of both hydrophobic and hydrophilic residues, and both of these processes may be working concurrently	(213)
Aqueous extract from *Piper longum* root	Antioxidant and anti-inflammatory activities	*Enterococcus faecium*, and *Acinetobacter baumannii*	Electrostatic interactions	(214)
Hexane extract from lemongrass leaves	Regulated hypertension, epilepsy, gastric, and central nervous system conditions	*Pseudomonas aeruginosa*, and *Enterobacter* species	Ionic interactions	(214)
Aqueous extract from *Aloe* leaves	Helped heal wounds, irritations, and scorching-related skin injuries; actively repaired damaged skin	*Pseudomonas aeruginosa*, and *Enterococcus casseliflavus*	Amphipathicity is the existence of both hydrophobic and hydrophilic residues, and both processes can be active at the same time	(214)
Ethanol extract from Bermuda grass leaves	Used as purgative, brain and heart tonic, coolant	*Staphylococcus aureus*	Electrostatic interactions	(214)
Methanol extract from cocoa seeds	Lowered blood pressure, healed damaged skin, and stimulated the nervous system. Beneficial for the treatment of anemia, diarrhea, and bruises	*Klebsiella pneumoniae*	Ionic interactions	(215)
Methanol extract from sweet potato leaves	Treatment for diabetes, hypertension, gastrointestinal problems, arthritis, rheumatic diseases, meningitis, renal conditions, and inflammations	*Klebsiella pneumoniae*	Amphipathicity denotes the coexistence of hydrophobic and hydrophilic residues, with both processes potentially occurring concurrently	(215)
Methanol extract from neem tree bark’s	Cured skin ailments, malaria, fevers, dental and gastrointestinal problems, and served as an insect repellent	*Enterobacter* species	Electrostatic interactions	(215)
Methanol extract from Pomelo leaves	Epilepsy, chorea, convulsive cough, and bleeding sickness treatment	*Enterobacter* species	Ionic interactions	(215)
Methanol extract from winter squash beans	Treatment for kidney problems and intestinal infections in addition to fighting tapeworms	*Enterobacter* species, and *Klebsiella pneumoniae*	Amphipathicity denotes the coexistence of hydrophobic and hydrophilic residues, with both processes potentially occurring concurrently	(215)
Methanol extract from bush butter tree leaves and seeds	Tonsillitis treatment by gargling and mouthwash	*Klebsiella pneumoniae*	Electrostatic interactions	(215)
Methanol extract from okra leaves	Nose and throat infections, urine issues, and gonorrhea treatment	*Klebsiella pneumoniae*	Ionic interactions	(215)
Methanol extract from common bean leaves	Treat obesity and diabetes	*Enterobacter* species, and *Enterococcus casseliflavus*	Amphipathicity denotes the coexistence of hydrophobic and hydrophilic residues, with both processes potentially occurring concurrently	(215)
Methanol extract from *Lantana* leaves	Reduce inflammation, treat malaria, relieve spasms, inhibit tumor growth, and prevent ulcer formation.	*Enterobacter* species, and *Pseudomonas aeruginosa*	Electrostatic interactions	(216)
Ethanol, hexane, and aqueous extract from Palash leaves	Stimulated diuresis and catamenial flow	*Enterobacter* species	Ionic interactions	(216)
Ethanol extract from burflower tree, red leaves, and sandalwood and bark	Treatment of fever, uterine difficulties, skin disorders, and inflammation. Febrifuge, antidiarrheal, and antihyperglycemic	*Acinetobacter baumannii*, and *Pseudomonas aeruginosa*	Amphipathicity denotes the coexistence of hydrophobic and hydrophilic residues, with both processes potentially occurring concurrently	(216)
Chloroform extract from *Andrographis paniculata* (green chireta)	Demonstrated efficacy in treating hypertension, ulcers, respiratory ailments, and dermatological issues while also exhibiting anti-cancer and anti-diabetic properties	Methicillin-resistant *Staphylococcus aureus* (MRSA)	The hydrophobicity of the plant extract may affect the reaction’s outcome when it interacts with the hydrophobic groups in the bacterial membrane	(216)
Methanol extract from stiff bottlebrush leaves	Diarrhea, dysentery, rheumatism, bronchitis, and cough cure	Methicillin-resistant *Staphylococcus aureus* (MRSA)	Amphipathicity refers to the existence of both hydrophobic and hydrophilic residues, and both of these processes can work simultaneously	(216)
Alcoholic extract from *Myrtle* leaves	Cancer, inflammations, diabetes, ulcers, hypertension, dysentery, rheumatism, and dysentery treatment	*Staphylococcus aureus*, and *Pseudomonas aeruginosa*	Electrostatic interactions	(216)
Aqueous extract from cinnamon bark	Demonstrated properties that combat diabetes, reduced oxidative stress, and alleviated inflammation	*Staphylococcus aureus*, and *Pseudomonas aeruginosa*	Ionic interactions	(217)
Aqueous extract from mint leaves	Displayed efficacy against acid reflux, irritable bowel syndrome, and the common cold	*Staphylococcus aureus*, and *Pseudomonas aeruginosa*	Amphipathicity refers to the existence of both hydrophobic and hydrophilic residues, which can work simultaneously	(217)
Aqueous extract from henna leaves	Treated burns and wounds, skin infections. Improved the health of hair	*Staphylococcus aureus*, and *Pseudomonas aeruginosa*	Electrostatic interactions	(217)
Aqueous extract from *Aloe* leaves	Digestive issues and burns treatment	*Staphylococcus aureus*, and *Pseudomonas aeruginosa*	Ionic interactions	(217)
Aqueous extract from ginger roots	Anti-cancer, vomiting, and nausea treatment	*Staphylococcus aureus*, and *Pseudomonas aeruginosa*	Amphipathicity refers to the existence of both hydrophobic and hydrophilic residues, which can work simultaneously	(217)
Chloroform extract from snake flower leaves and bulbs	Skin and wound infection treatment	*Staphylococcus aureus*, and *Pseudomonas aeruginosa*	Hydrophobicity of plant extract	(217)
Chloroform extract from bitter *Aloe* leaves	Skin disorders treatment	*Staphylococcus aureus*, *Pseudomonas aeruginosa*, and *Acinetobacter baumannii*	The plant extract binds to the bacterial membrane’s hydrophobic groups	(219)
Ethanol extract from horse mint aerial parts	Alleviated irritation of the throat, mouth, and sore throat	*Mycobacterium tuberculosis*	Amphipathicity denotes the coexistence of hydrophobic and hydrophilic residues, with both processes potentially occurring concurrently	(219)
Ethanol extract from amla aerial parts	Anti-diabetic, antioxidant, anti-inflammatory	*Mycobacterium tuberculosis* MRSA	Electrostatic interactions	(220)
Dichloromethane extract from *Aloe* leaves	Treated skin, gastrointestinal, and respiratory disorders	*Staphylococcus aureus*, and *Pseudomonas aeruginosa*	Plant extract hydrophobicity	(220)
Methanol extract from cardamom fruit	Employed as a replacement drug for the local people and for scientific research to find new medications to address the problems caused by the increasing prevalence of antibiotic resistance	*Staphylococcus aureus*	The ionic and electrostatic interactions result in the plant extract deposition on the bilayer’s surface, leading to instability and disintegration of the cellular membrane	(221)

### 7.1 Disruption and inhibition of microbial cell membranes

Numerous bioactive chemicals in medicinal plants, including terpenoids, flavonoids, and alkaloids, engage with microbial cell membranes, resulting in structural impairment. These chemicals can enhance membrane permeability, resulting in the efflux of cellular constituents and subsequent cell death (173, 182, 208). Compounds such as carvacrol (derived from oregano) and thymol (extracted from thyme) compromise the lipid bilayer of bacterial membranes, undermining their structural integrity (173, 208).

Moghrovyan and Sahakyan (246) revealed that essential oils derived from *Origanum vulgare* compromised the membrane potential of *E. coli* and *S. aureus*, resulting in cell lysis (246). Alkaloids derived from *Dicranostigma leptopodum* enhance the permeability of *K. pneumoniae* cells, impeding their development (246). Due to their lipophilic characteristics, terpenes easily infiltrate bacterial cell membranes, resulting in damage and demonstrating antibacterial or bactericidal properties, as evidenced by carvacrol, thymol, and eugenol against several bacterial strains (247). Flavonoids similarly compromise the cell membranes of *Candida* species, increasing permeability and disrupting membrane protein functionality (247). Furthermore, the alkaloid tomatidine, present in potatoes, inhibits the enzymes involved in ergosterol production in many species of *Candida* and *Saccharomyces* (248).

Flavonoids exhibit potential as antimicrobials by blocking bacterial fatty acid biosynthesis enzymes, such as quercetin, apigenin, and sakuranetin from *Plukenetia volubilis* leaves, which target specific enzymes in *H. pylori* (249). These flavonoids, such as quercetin and apigenin, inhibit peptidoglycan production, an essential component of the bacterial cell wall (249). In fungi, acidic terpenoids such as enfumafungin block β (1, 3)-D-glucan synthase, an enzyme essential for glucan production in the cell wall, by a non-competitive mechanism (250). Furthermore, the flavonoid glabridin compromises the structural integrity of fungal cell walls, illustrating an alternative mechanism for antifungal action (251).

### 7.2 Inhibition of efflux pumps

Plant-derived chemicals provide many techniques to address microbial resistance, especially via inhibiting efflux pumps (252). Efflux pumps constitute a primary mechanism of antibiotic resistance in MDR bacteria. Medicinal herbs possess chemicals that can obstruct these pumps, so limiting the evacuation of antibiotics and augmenting their effectiveness (252). The many methods, such as membrane rupture, interference with biosynthesis and metabolism, biofilm inhibition, and reactive oxygen species formation, underscore the potential of plant-derived chemicals, particularly those from the Caucasus region, to address antimicrobial resistance (252). Flavonoids and alkaloids, including berberine (derived from *Berberis* species), interact with efflux pump proteins, inhibiting their activity (182, 208, 252).

Yu et al. (252) emphasized that berberine blocked the NorA efflux pump in MRSA, hence reversing resistance to ciprofloxacin. Alkaloids, including ellagic and tannic acids, together with different flavonoids, impede efflux pump activity in bacteria such as *A. baumannii* (253). Terpenes, notably carvacrol, serve as effective inhibitors of efflux pumps, particularly in *S. aureus*, possibly via influencing gene expression or binding to efflux proteins (254). Curcumin, a polyphenol, regulates efflux pump activity in yeast, whereas many flavonoids exhibit comparable inhibitory effects in fungi, resulting in cellular mortality (254).

### 7.3 Inhibition of biofilm formation

Biofilms are protective matrices created by MDR microorganisms, rendering them resistant to antibiotics. Compounds derived from medicinal plants can impede biofilm growth or dismantle pre-existing biofilms (255). Polyphenols and tannins disrupt quorum sensing, a critical communication mechanism for biofilm development (255). Flavonoids and alkaloids exhibited notable antibiofilm efficacy against several microbial species. Flavonoids, including phloretin, can impede biofilm development in *E. coli* by downregulating curli gene expression, hence limiting fimbriae formation (255). They can also elicit aberrant multicellular aggregation in *S. aureus*, inhibiting the formation of structured biofilm formations (155). Alkaloids such as anguinarine and berberine derived from apple significantly inhibit biofilm formation in *Proteus rettgeri* and MDR *E. coli*, respectively, by disrupting the synthesis of biofilm-related compounds (256).

Moreover, some flavonoids (quercetin, apigenin) and phenolic acids (ellagic, caffeic) impede biofilm formation in *Candida* species, including the drug-resistant *Candida auris* (257). Prenyflavanone 8PP inhibits *C. albicans* biofilms by enhancing the generation of reactive oxygen and nitrogen species (257). Curcumin from *Curcuma longa* suppresses biofilm formation in *P. aeruginosa* via downregulating quorum sensing-related genes (258).

### 7.4 Interference with microbial enzymes

Compounds from medicinal plants can inhibit essential enzymes critical for microbial survival, including DNA gyrase, topoisomerase, and β-lactamase. Alkaloids and phenolic acids attach to the active sites of these enzymes, hindering DNA replication and cellular division (259). Bioactive phytochemicals interfere with bacterial metabolism via multiple methods. Bioflavonoids can impede the F1 or membrane-associated F1Fo ATPases of *E. coli* (259). Catechin and epigallocatechin gallate inhibit F1Fo-ATPase and lactate dehydrogenase in *Streptococcus mutans* (259). Flavonoids derived from *Chimonanthus salicifolius* SY also influence ATPase activity in *P. aeruginosa* and *S. aureus*, impeding their development (259).

Berberine derived from *Corydalis turtschaninovii* modifies glucose metabolism in *Streptococcus pyogenes* via regulating carbohydrate absorption and transformation (260). Moreover, terpenes have antifungal properties against several fungi by obstructing respiratory enzymes such as succinate dehydrogenase and NADH oxidase. Moreover, epigallocatechin gallate (EGCG) derived from green tea decreased β-lactamase activity in *K. pneumoniae*, hence reinstating susceptibility to β-lactam drugs (261).

### 7.5 Induction of oxidative stress

Numerous plant-derived chemicals, including quinones and polyphenols, produce reactive oxygen species in microbial cells, surpassing their antioxidant defenses and inducing oxidative damage (262). Reactive oxygen species inflict damage on DNA, proteins, and lipids, resulting in cellular apoptosis (262). The antibacterial efficacy of catechin against Gram-positive and Gram-negative bacteria is ascribed to the induction of reactive oxygen species subsequent to membrane permeabilization. Likewise, berberine induces elevated reactive oxygen species generation in *S. pyogenes* (262).

In *Candida* species, the flavonoid baicalein induces reactive oxygen species buildup and consequent death. Moreover, honey flavonoids and quercetin impede the growth of *C. albicans* by increasing intracellular reactive oxygen species levels (263). Santra et al. (264) demonstrated that plumbagin, derived from *Plumbago zeylanica*, produced oxidative stress in *C. albicans*, leading to fungal cell death (264).

### 7.6 Modulation of host immune responses

Certain medicinal plants augment the host immune system, enhancing its capacity to combat MDR microorganisms. Polysaccharides and saponins activate immune cells, including macrophages and neutrophils, to phagocytize infections (265). Natural immunostimulants, such as probiotics and traditional Chinese medicines, are progressively utilized as animal feed additives to manage *Salmonella pullorum* infections, owing to their environmentally favorable characteristics and absence of chemical residues (265). *Zanthoxylum bungeanum* meal, a byproduct of oil production, possesses bioactive chemicals and exhibits extensive antibacterial activity (265).

Likewise, *Schisandra chinensis* and *Scutellaria baicalensis* exhibit anti-inflammatory, antioxidant, and antibacterial characteristics and are recognized for their ability to improve animal development and productivity (265, 266). Conversely, Abdelmotaleb et al. (267) reported that *Echinacea purpurea* extracts augmented macrophage function against MDR *M. tuberculosis*.

### 7.7 Synergistic effects with conventional antibiotics

Herbal medicines frequently demonstrate synergistic effects when used in conjunction with antibiotics, hence augmenting their effectiveness against MDR pathogens (208, 268). Phytochemicals can diminish microbial defenses, rendering them more vulnerable to drugs. Hasna et al. (268) examined the synergistic effects of methanolic extracts from four Algerian medicinal plants (garlic, red onion, cumin, and fenugreek) in conjunction with several strains of lactic acid bacteria against *H. pylori*. The *in vivo* antibacterial efficacy of fenugreek extract in conjunction with *Bifidobacterium breve* was assessed to validate the synergistic impact of this particular combination on *H. pylori* colonization (268). All evaluated combinations of plant extracts and probiotics exhibited inhibitory activity against *H. pylori*, but with differing degrees of efficacy (268).

Saquib et al. (269) examined the synergistic antibacterial properties of ethanolic extracts from *Punica granatum* (pericarp), *Commiphora molmol*, and *Azadirachta indica* (bark) in conjunction with standard antibiotics (amoxicillin, metronidazole, tetracycline, and azithromycin) against principal periodontopathic bacteria (*Porphyromonas gingivalis*, *Tannerella forsythia*, *Treponema denticola*, and *Aggregatibacter actinomycetemcomitans*). Synergistic antibacterial action was noted when plant extracts were coupled with antibiotics, with the most effective synergy observed between *P. granatum* extract and amoxicillin against *A. actinomycetemcomitans* (269). Magryś et al. (270) observed that the combination of *Allium sativum* (garlic) extract with amoxicillin considerably decreased the MIC against MDR *H. pylori*.

Ayaz et al. (271) conducted a study to investigate the antibacterial efficacy and minimum bactericidal concentration (MBC) of three particular agents (thymol, EDTA, and vancomycin) against both susceptible and resistant bacterial strains. The research also examined the synergistic effects of thymol and EDTA, as well as the interactions of vancomycin with these two compounds (271). Thymol exhibited significant bactericidal activity, with MBC between 60 and 4000 g ml/1. Conversely, EDTA exhibited bacteriostatic activity solely beyond the MBC range of 60-4000 g ml/1 (271).

Gram-positive bacteria demonstrated MICs between 0.125 and 16 g ml/, whereas Gram-negative bacteria displayed MICs from 32 to 128 g ml/1. The interaction mechanism between various chemical combinations and MRSA and *E. coli* was examined by checkerboard dilution and time-kill curve assays (271).

### 7.8 Inhibition of virulence factors

Medicinal plants can inhibit the synthesis of virulence components, including toxins and adhesion molecules, hence diminishing the pathogenicity of MDR microorganisms (208, 272). Compounds such as resveratrol and quercetin inhibit the expression of genes that encode virulence factors. Resveratrol, derived from grapes, suppressed the synthesis of staphylococcal enterotoxins in MRSA (272). El-Mahdy et al. (273) suggested that resveratrol and curcumin may serve as alternative treatment strategies for treating MRSA infections by diminishing toxin production.

## 8 Medicinal plants as a complementary therapy

Humans and animals have used medicinal plants to treat a variety of diseases (274). Nonetheless, understanding of the causes of diseases and the curative qualities of specific plants remained severely limited; all information was based on empirical experimentation. Following the acquisition of suitable knowledge, transactions were based on insights gained via vast experience (274, 275). Prior to the advent of iatrochemistry in the 16^th^ century, these herbs served as a means of treatment and prophylaxis (275, 276). The practice of Ayurvedic medicine, which demonstrates the healing properties of plants, indicates that old approaches have not been forgotten despite the introduction of antibiotics (275).

Although not all plants display these traits, the primary bioresource of active chemicals is found in esteemed medicinal herbs (277, 278). Various established bioactive chemicals have been employed in Ayurvedic practices, modern medicine, nutraceuticals, and pharmaceutical intermediates, serving as lead molecules for synthetic drugs and are currently accessible for purchase (277–279). The function of these molecules in development, photosynthesis, reproduction, or other fundamental activities remains undetermined. Secondary metabolites are synthesized to defend against biotic and abiotic stressors, including fungal and bacterial infections (280). Since antiquity, botanical products have served as the principal resource for human welfare and the cornerstone of all life on Earth (278, 280). Karunamoorthi et al. (281) and Jamshidi-Kia et al. (282) indicated that humans have utilized plants with medicinal properties as the foundation of intricate traditional medicine to treat diseases worldwide.

The utilization of medicinal plants can be dated to antiquity through various historical texts (283, 284), for example, records from Ancient Egypt date to 1500 BCE, the Chinese medical text “Shen Nong Ben Cao” originates from 200 CE, and Dioscorides’ De Materia Medica chronicles the Mediterranean pharmacopeia between 50 and 70 CE (283). WHO estimates indicate that 80% of individuals in poor nations primarily rely on traditional herbal remedies for their medicinal needs (284).

Research undertaken from 1981 to 2007 on the development of new pharmaceuticals, including antimicrobials, indicated that over 50% of the drugs licensed since 1994 are derived from natural sources (23, 285, 286). It is estimated that almost 80% of the chemicals utilized in pharmaceuticals globally are derived from natural sources, and now, there are more than a 100 novel candidates based on raw materials in the clinical development phase (285, 286). Moreover, despite substantial advancements in chemical synthesis, at least 25% of the medications currently utilized in contemporary medicine may be traced back to plant roots (286). This encompasses 60% of anticancer therapy and 75% of antiinfective medicines (287).

The choice of plant species is essential for the success of novel anti-infective discoveries in pharmaceutical development. Random selection, chemotaxonomic, and ethnopharmacological methodologies are employed to identify plants for biological and phytochemical investigation (288). The indiscriminate method sometimes entails collecting plants from several regions to generate a substantial quantity of plant samples, ensuring considerable chemical diversity and enhancing the likelihood of discovering a biologically active component (289). The sample of plant components is frequently selected and examined without the benefit of ethnobotanical and chemotaxonomic expertise (288). The presence of certain chemotaxonomic markers within each family or genus is a determinant in the selection of plants utilizing the chemotaxonomic method (289). This technique entails phytochemical screening to detect particular active biological markers in the plant, including flavonoids and alkaloids. The foundation for selection and evaluation in the ethnopharmacological method is the oral or written knowledge of a plant’s historic therapeutic application (290).

Ethnopharmacology is a versatile approach for discovering novel antibiotics. This subject is an interdisciplinary domain that integrates diverse fields like botany, chemistry, biochemistry, pharmacology, anthropology, archeology, history, and languages (290, 291). It specifically emphasizes the systematic observation, description, and experimental investigation of traditional medicine and the traits that hinder organism development (290, 291). Numerous resources exist for studying organized conventional medicinal systems, such as traditional Chinese medicine, Unani, Kampo, and Ayurveda. These resources encompass computer databases, fieldwork, literature, herbals, and review articles that frequently provide evaluations of medicinal plants according to geographic region or ethnic culture (291).

This approach has demonstrated remarkable efficacy in screening and identifying plants containing bioactive compounds potentially useful for the development of anti-infective pharmaceuticals (290). The technique involves choosing plant species according to the preferences of specific demographic groups for the treatment of infectious diseases (e.g., diarrhea, malaria) (291). The selected plants will be subjected to extraction processes, such as the acquisition of a crude extract or essential oil, which will then be evaluated for their ability to inhibit the growth of harmful microorganisms (290, 291).

Following the confirmation of the extract’s inhibitory activity, characterized by dose dependence (MIC), a targeted assay may be performed to identify the active constituents (292). This procedure entails the selection and refinement of the most active fraction(s) (293). Chemical testing methodologies and contemporary pharmacognosy are employed to ascertain the composition of the final fraction. The identified chemicals may be utilized to test and validate biological targets specific to disease (292, 293).

Researchers evaluated almost 2000 traditional Chinese herbal formulas derived from ancient texts of traditional Chinese medicine (294). Clinical investigations demonstrated that the ether extract of *A. annua* effectively inhibits the growth of *Plasmodium falciparum*, the causative cause of malaria (294). Subsequently, the researchers synthesized the derivative dihydroartemisinin and isolated the pure component artemisinin (294). The WHO advocates for artemisinin-based combination therapy as the primary treatment for malaria, which is extensively utilized worldwide (22).

Traditional Chinese medicine uses the roots and rhizomes of *Coptidis* spp. (Ranunculaceae) to treat ailments such as dysentery and diarrhea attributed to damp heat. The Chinese Pharmacopeia identifies the three Coptis species constituting “Huanglian” as Coptis chinensis, Coptis deltoidea, and Coptis teeta. Qi et al. (295) assert that Huanglian comprises berberine, an isoquinoline alkaloid, which is the principal constituent accountable for its antidiarrheal effects (295).

Numerous researchers have delineated the MIC range of berberine against diarrhoea-associated bacteria, such as *S. aureus*, MRSA, *Vibrio cholera*, *Bacillus cereus*, and *E. coli*, as 12.5–469 µg/ml (296–298). Numerous clinical investigations including pediatric patients have demonstrated that berberine and its derivatives (berberine tannate, hydrochloride, and berberine sulfate) are useful in treating diarrhea. Acute infectious diarrhea was effectively managed with berberine tannate and its combination with sulfadimidine and neomycin, as reported by Khameneh et al. (243). Clinical studies have demonstrated that berberine tannate is efficacious, including in pediatric patients with gastroenteritis (299). Research indicates that berberine hydrochloride and berberine sulfate effectively treat diarrhea in comparison to conventional antibiotics like chloramphenicol and streptomycin. They can significantly diminish the volume of liquid stool and the frequency of urgent bowel motions (300, 301).

Several pharmaceutical companies have promoted berberine as a therapeutic agent for diarrhea and intestinal infections (302). It is obtainable as an extract, exemplified by Huang Lian Su Tablets derived from *Coptis chinensis* extract, or in its pure form, such as berberine hydrochloride tablets produced by Northeast Pharmaceutical Group in Shenyang, China (302). A further example of effective ethnopharmacological research is the extraction of essential oil from *Melaleuca alternifolia* (Myrtaceae), a procedure equally grounded in ethnopharmacological principles. The tree is a shrub with delicate, parchment-like bark native to the humid coastal regions of northern New South Wales, Australia (302). The Bundjalung Aboriginal people utilized leaf poultices on wounds and ailments, inhaling vapors from burned leaves to alleviate coughs and colds (302). Numerous antimicrobial studies have demonstrated that tea tree oil exhibits antibacterial activity against *S. aureus* and MRSA in both *in vitro* and human trials (277). Numerous monoterpenes elucidate its inhibitory impact, including 1,8-cineole, β-terpinene, and terpinene-4-ol (302). Tea tree oil has been extensively marketed and utilized in inhalation therapy for the treatment of bacterial and fungal pneumonia (303).

Notwithstanding these facts, research on the screening, identification, and characterization of plant constituents is still regarded as crucial for discovering novel phytochemicals, particularly those with antimicrobial activities (304, 305). The work is ongoing, and further plants with antibacterial actiivites have been uncovered, including garlic (306), cinnamon (307), *Glycyrrhiza glabra* (liquorice), *Viola odoratta*, *Amomum subulatum*, *Elettaria cardamomum* (308), among numerous others. Thus, it indicates considerable potential for the development of new antibacterial agents from underexploited plants (309). This assertion is corroborated by other articles indicating that medicinal plants are the principal sources of antibacterial secondary metabolites (309). It is important to note that hardly a minuscule percentage of the estimated 750,000 plant species on Earth have been studied and utilized (310).

## 9 The long-term use of medicinal plants and the potential microbial resistance

For generations, medicinal plants have been utilized to address numerous ailments. Nonetheless, the prolonged utilization of these plants may foster the emergence of microbial resistance (311). Microbial resistance refers to the capacity of microorganisms, including bacteria, fungi, viruses, and parasites, to withstand the effects of medications that were previously effective in treating infections caused by these organisms (312). This resistance can complicate infection treatment, resulting in prolonged illnesses, elevated healthcare expenses, and sometimes fatal outcomes (311, 312).

The excessive usage and improper use of therapeutic herbs might generate selective pressure that promotes the survival of resistant bacteria. This parallels the phenomenon where excessive antibiotic use results in antibiotic resistance. Nakamoto et al. (313) asserted that garlic possesses several chemicals exhibiting antibacterial properties. Nonetheless, excessive use of garlic may result in the emergence of resistance to these chemicals. *Echinacea* (coneflower) is a widely utilized herbal treatment for colds and influenza. Nevertheless, research indicates that prolonged usage of *Echinacea* may diminish the efficacy of the immune system (313, 314).

Additionally, *Hydrastis canadensis* (goldenseal) comprises a component known as berberine, which exhibits antimicrobial properties. Excessive use of goldenseal may result in the development of resistance to berberine (314). Conversely, sub-lethal doses of medicinal herbs may fail to eradicate all microorganisms responsible for infection. The remaining organisms may subsequently acquire resistance to the medicinal plant (315). Moreover, resistance genes can be disseminated among microorganisms via other means, including plasmids and transposons. This indicates that a microorganism can obtain resistance genes from other bacteria, even without direct exposure to a medicinal plant (316, 317). Not all therapeutic herbs contribute to microbial resistance. Certain medicinal herbs may aid in the prevention of resistance development (318). Research indicates that cranberry juice may aid in the prevention of urinary tract infections by inhibiting bacterial adhesion to the urinary tract walls (319).

### 9.1 Factors influencing the development of resistance

In contrast to single-molecule antibiotics, medicinal plants comprise a complex array of bioactive chemicals, such as alkaloids, terpenoids, flavonoids, phenolics, and others. The “cocktail” effect poses a distinct challenge to bacteria striving to acquire resistance (320). Unlike conventional medicines that typically target a singular bacterial route, plant extracts frequently demonstrate antimicrobial activity via many pathways (320). This complex assault substantially complicates the ability of microorganisms to evolve resistance, as they are required to create several resistance mechanisms simultaneously (320). Several factors influence the development of resistance (320), as outlined below:-

- Complexity of the extract: A higher diversity of bioactive chemicals in the plant extract correlates with reduced selective pressure for resistance. A diverse array of chemicals that target various biological systems complicates the adaptation of microorganisms (320).
- Concentration of active compounds: The conventional application of medicinal plants typically entails lesser amounts of active constituents compared to pure antibiotics. Reduced concentrations can diminish selective pressure, when microorganisms encounter sublethal dosages that may impede growth without causing mortality, so facilitating adaptation (320, 321). It is crucial to acknowledge that certain plant extracts may include elevated levels of certain chemicals, thereby intensifying selective pressure (321).
- Duration and frequency of exposure: Prolonged and repeated exposure to plant extracts may elevate the chance of resistance development, even at low dosages. Extended exposure offers increased opportunity for microorganisms to adapt and evolve (320, 321).
- Specific compounds versus whole extracts: Resistance is more probable to emerge against isolated plant chemicals, particularly when utilized as single-molecule antibiotics. The utilization of entire plant extracts, characterized by their intricate compositions, is typically seen as presenting a diminished risk of resistance (320).
- Microbial species: Diverse microbial species possess differing abilities to acquire resistance. Certain species possess more adaptability and a higher propensity for developing resistance than others.
- Presence of resistance genes: Microorganisms can obtain resistance genes via horizontal gene transfer. The existence of pre-existing resistance genes within a microbial community can elevate the probability of resistance emergence against plant-derived antimicrobials, primarily if these compounds target the exact mechanisms as traditional antibiotics (316, 317).

## 10 Future concerns

The potential for employing medicinal plants against MDR bacteria is significant; nonetheless, it requires thorough research and development. Despite the extended history of medicinal plant utilization, there is a significant gap in our understanding of the sustained evolution of resistance to these complicated combinations. Research should focus on investigating the effects of extended exposure to medicinal plant extracts on microbial communities; determining the specific mechanisms by which bacteria may develop resistance to plant-derived antimicrobials; assessing the rate of resistance emergence to botanical extracts relative to conventional antibiotics; establishing standardized methodologies for evaluating antimicrobial efficacy and resistance emergence to plant extracts; and monitoring for signs of reduced efficacy of herbal remedies in clinical settings.

### 10.1 Unveiling the potential of medicinal plants

Medicinal plants contain a substantial array of bioactive chemicals with various modes of action. These chemicals may potentially surmount the resistance mechanisms established by MDR bacteria. The intricate amalgamation of phytochemicals in medicinal plants can demonstrate synergistic effects, wherein the collective activity surpasses the sum of its individual constituents. This combination can augment antibacterial efficacy and diminish the probability of resistance emergence.

### 10.2 Overcoming challenges

Maintaining uniform quality and effectiveness of plant-derived pharmaceuticals is essential. This necessitates uniform growing procedures, extraction techniques, and quality control protocols to ensure the consistency of active chemicals. Comprehensive scientific research is essential to confirm the effectiveness and safety of therapeutic plants against MDR bacteria. This includes *in vitro* and *in vivo* investigations to ascertain the mechanisms of action, ideal doses, and possible toxicity. Furthermore, the overexploitation of therapeutic plants may result in their depletion. Sustainable harvesting methods and conservation initiatives are crucial for guaranteeing the enduring availability of these precious resources.

### 10.3 Future directions

Medicinal plants can provide innovative lead chemicals for the development of new antibiotics. Advanced methodologies like genomics, proteomics, and metabolomics help expedite the identification and characterization of bioactive compounds. Integrating plant-derived antimicrobials with current antibiotics can augment their efficacy and address resistance. This method can help decrease the dosage of traditional antibiotics, hence reducing adverse effects and the emergence of further resistance. Comprehending the interplay between phytochemicals and specific patients can facilitate tailored therapeutic approaches. This method considers personal genetic composition, gut microbiota, and additional variables to enhance the efficacy of phytotherapeutics.

### 10.4 Cooperation and knowledge exchange

Combining traditional knowledge of medicinal plant usage with contemporary scientific studies can yield significant insights into their effectiveness and safety. Collaboration among scientists, healthcare practitioners, and traditional healers is crucial for converting research findings into applicable clinical practices. Moreover, exchanging information and resources among nations helps expedite the discovery and development of novel plant-based pharmaceuticals to address MDR bacteria globally. By confronting the problems and seizing the opportunities, medicinal plants can significantly contribute to combating MDR bacteria, providing a sustainable and potentially transformative healthcare solution.

## 11 Conclusion

Medicinal plants serve multiple purposes due to their efficacy, few side effects, and phytochemical constituents that can effectively treat numerous ailments. Utilizing potent medicinal herbs to address specific ailments mitigates or diminishes infections. Approximately 85% of the populace in developing nations utilizes traditional medicine, predominantly herbal medicines. Addressing antibiotic resistance is a crucial global health concern. This review examines research from 2014 to 2025 regarding the potential of medicinal plants as sources of novel antimicrobial agents. Due to the escalating issue of antibiotic resistance, these plant extracts present viable alternatives to traditional antibiotics, which are progressively losing efficacy.
